# Differential perception of virulence factors of uropathogenic *Escherichia coli* at the level of chromatin dynamics of infected host cells

**DOI:** 10.3389/fimmu.2025.1642683

**Published:** 2025-10-06

**Authors:** Krishnendu Mukherjee, Wiebke Aschenbach, Annika Hilger, Judith Saur, Ulrich Dobrindt

**Affiliations:** Institute of Hygiene, University of Münster, Münster, Germany

**Keywords:** insect immunity, UPEC, pathogenicity islands, Galleria mellonella, epigenetics, innate immunity

## Abstract

**Introduction:**

Uropathogenic *Escherichia coli* (UPEC) evades the innate immune response in the urinary tract through the coordinated action of various virulence factors encoded within distinct pathogenicity islands (PAIs). We have demonstrated that UPEC infection leads to the epigenetic regulation of host gene expression; however, the specific role of PAI-encoded virulence factors in this process remains largely unexplored.

**Methods:**

In this follow-up study, we infected *Galleria mellonella* larvae with individual PAI deletion mutants of UPEC strain 536 to investigate the relationship between UPEC virulence determinants and host epigenetic regulation.

**Results:**

The loss of different pathogenicity islands (PAI I_536_ to PAI VI_536_) led to varying degrees of virulence attenuation in larvae and an increased sensitivity to *G. mellonella* hemolymph compared to the wild-type UPEC strain 536. Notably, infection with the different PAI mutants resulted in distinct histone modification patterns, including hypo- or hyper-acetylation of specific histone H3K9 and H4K5 residues. In addition, the loss of selected PAIs led to altered expression of histone acetyltransferases and histone deacetylases as well as changes in the expression of antimicrobial innate immune genes. We show that UPEC-induced histone acetylation changes in larvae were conserved in human bladder epithelial cells, underscoring the translational relevance of the *G. mellonella* system.

**Discussion:**

These findings reveal that specific PAI-encoded virulence factors trigger epigenetic and immunological changes in *G. mellonella* which may help us to also better understand relevant processes in the course of infection in humans.

## Introduction

Pathogenicity islands encode many fitness traits that improve the adaptability of pathogenic bacteria to different host environments compared to non-pathogenic variants. A classical example of this is uropathogenic *Escherichia coli* (UPEC) whose genomes contain different pathogenicity islands (PAIs) that can help them to colonize the normally sterile urinary tract of humans (1–4). PAIs enable UPEC to evade the host’s immune response as well as antimicrobial therapies, establishing it as the primary cause of over 75% of uncomplicated urinary tract infections (UTIs) diagnosed annually in humans (5, 6). PAIs encode a range of virulence factors, including adhesins, toxins, capsules, specific O antigens, and iron-uptake systems, enabling UPEC to cause symptomatic UTIs (4, 7). In contrast, inactivation of virulence factors through reductive evolution can enable certain UPEC strains to adapt to a commensal-like lifestyle for long-term persistence within the urinary tract, leading to asymptomatic bacteriuria (ABU) (8, 9). The role of selected UPEC virulence factors has been investigated in isolation, but the possible interplay and interdependencies between individual virulence factors in the context of perception by the innate host defenses remain poorly understood. Establishing the link between virulence and the role of individual PAIs in shaping host response to UPEC infection demands a thorough investigation. From an evolutionary perspective, it remains to be determined whether the loss of selected PAIs can lead to compromised UPEC virulence and influence the molecular mechanisms governing host response to infection, such as the expression of key innate immunity-related genes. Gaining such insights is crucial for understanding UPEC adaptation strategies as well as identifying novel disease markers and therapeutic targets for UTIs.

In eukaryotes, gene expression is regulated by epigenetic mechanisms like histone acetylation resulting in heritable phenotypic alterations independent of DNA sequence mutations (10). Histone acetyltransferases (HATs) and histone deacetylases (HDACs) regulate the transfer of acetyl groups to and from histones, exhibiting opposing functions. HATs acetylate histones, enhancing DNA accessibility and promoting gene expression, while HDACs remove acetyl groups, reducing DNA accessibility and suppressing gene expression. Many human pathogenic bacteria can produce effector molecules that disrupt the balanced regulation of HDACs and HATs, affecting histone acetylation (11, 12). The Gram-positive bacterium *Listeria monocytogenes* produces listeriolysin-O (LLO) to deacetylate histones (13). Similarly, the Gram-negative bacterium *Anaplasma phagocytophilum* produces ankyrin A to target HDAC1, while enteropathogenic *Escherichia coli* produces the zinc-dependent metalloproteinase NleC to degrade HAT p300 in an infected host (14, 15). UPEC secretes α-hemolysin to inhibit the expression of proinflammatory cytokine genes by reducing histone acetylation in cell culture (16). Although the role of individual PAIs in UPEC pathogenesis was demonstrated in murine urinary tract infection models, there is limited information on the connection between UPEC virulence and epigenetic changes in an infected host (17). Moreover, analyzing the individual PAIs in the mouse model requires investigations with large sample sizes, which are likely to raise both ethical and economic concerns (18). Furthermore, the unavoidable cross-talk with adaptive immunity complicates the study of only innate immune responses in mice, highlighting the need to develop alternative *in vivo* model systems.

Insects have become valuable alternative model hosts for studying human pathogens, offering several advantages, including their small size, cost-effective mass rearing, exemption from ethical concerns, and the presence of only an innate immune system. The larvae of the greater wax moth, *Galleria mellonella*, are widely used to investigate the virulence potential of human pathogens, including UPEC and other extraintestinal pathogenic *E. coli* (ExPEC) (19–22). The sequenced genome and transcriptome of *G. mellonella* facilitates focused investigations into innate immunity and its associated epigenetic mechanisms like histone acetylation that are conserved in humans (23–25). Additionally, the larvae can survive at 37°C, the temperature at which humans encounter UPEC. The *G. mellonella* infection model has revealed differences in the virulence of UPEC clones isolated from patients with community-acquired and nosocomial UTIs (19). More recently, we identified a potential link between histone acetylation and innate immune gene expression, which distinguishes host responses to uropathogenic and commensal *E. coli* strains in *G. mellonella* larvae (23). However, it remains to be determined whether *G. mellonella* can distinguish the individual contributions of different PAIs to UPEC pathogenesis in a way that reflects infection dynamics in the human bladder epithelium. This could offer new opportunities to investigate how UPEC virulence factors contribute to host epigenetic regulation during infection.

**Figure 1:**
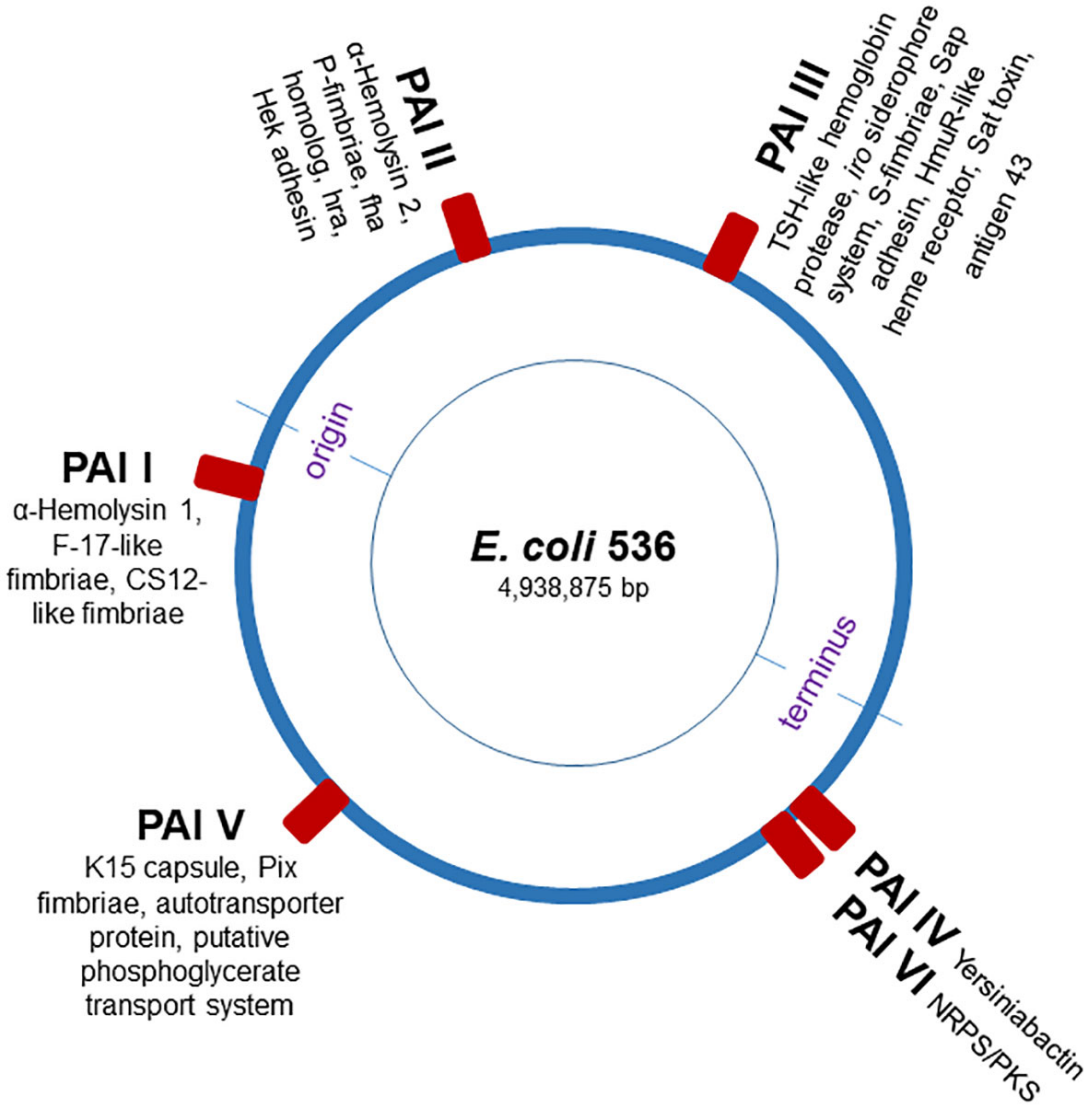
Schematic map of the *E. coli* strain 536 chromosome. The blue circle represents the *E. coli* chromosome, and red boxes indicate the pathogenicity islands (PAI I_536_ to PAI VI_536_), virulence factor-encoding genes [adopted from (17)].

Here, we utilized the *G. mellonella* larvae to investigate the role of individual PAIs in regulating conserved epigenetic mechanisms and innate immune responses to UPEC infection. The UPEC strain 536 (O6:K15:H31) is characterized by strong hemolytic activity and significant genomic differences in its PAIs compared to other UPEC strains, such as model strain *E. coli* CFT073 (17). The genome of UPEC strain 536 is well characterized and comprises many typical UPEC virulence-associated genes associated with archetypal PAIs (**Figure 1**). We systemically infected *G. mellonella* larvae with individual PAI deletion mutants of the UPEC strain 536. Our findings demonstrate that infection with individual PAI deletion mutants resulted in alteration of host responses at both the innate immunity and epigenetic levels, with similar patterns observed across other mammalian models. These results support our hypothesis that the contribution of individual PAIs extends beyond direct UPEC pathogenicity, influencing host responses at the epigenetic level.

## Materials and methods

### Bacterial strains, insects, and culture media

The bacterial strains used in this study are listed in **Supplementary Table 1**. The uropathogenic *E. coli* isolate 536 and many of its well-characterized PAI deletion mutants have already been published (17). Bacterial cultures were grown aerobically in lysogeny broth (LB) (Carl Roth, Germany) at 37°C and on LB agar plates. For long-term preservation, the bacteria were stored at −80°C in LB containing 30% glycerol. *Galleria mellonella* larvae were purchased from Fauna Topics Zoobedarf Zucht und Handels GmbH, Marbach am Neckar, Germany, and maintained as described previously (26).

### *G. mellonella* injection and homogenization

For injection experiments, we used bacterial cultures in the logarithmic growth phase in 3 ml of LB. The bacterial inocula were washed, serially diluted in 0.9% NaCl, and 10-μl aliquots of bacterial suspensions adjusted 1 x 10^6^ CFU/ml were injected into the larvae (1 x 10^4^ CFU/larva) in their sixth developmental stage (weight ~250 mg - 300 mg) through the left proleg using 1-ml disposable syringes with 0.4- by 20-mm needles mounted on a microapplicator. Larvae injected with an empty needle or 0.9% NaCl served as uninfected controls.

Larvae were considered dead after incubation at 37°C if they showed no movement in response to touch post-injection. Larvae injected with the wild type UPEC strain 536 (from now on UPEC strain 536) or the different PAI mutants (*E. coli* strain 536ΔPAI I – *E. coli* strain 536ΔPAI VI) were crushed to fine powder under liquid nitrogen at two different time points (8 h and 24 h post-injection). At each time point, three replicate samples were collected, each containing five whole larvae for RNA and histone isolation.

### Preimmune activation of *G. mellonella* larvae and antibacterial activity assays

The broth microdilution method was employed to assess the antibacterial activity of the *G. mellonella* hemolymph. Sixth-instar larvae were injected independently with heat-killed *E. coli* DH5α to trigger strong immune responses. The heat-killed preparation of *E. coli* DH5α was obtained as follows: An exponential-phase bacterial culture was harvested, centrifuged, and washed three times in 0.9% NaCl. The washed bacteria were resuspended in 0.9% NaCl and incubated at 85°C for 1 h. After two additional washes, the bacterial pellet was resuspended in 0.9% NaCl to a final concentration of 10 mg/ml. 10 μl of this suspension was injected directly into the hemolymph of the larvae to induce an immune response. Hemolymph samples from these larvae were extracted at 24 h following immune induction by the administration of the heat-killed *E. coli* DH5α. Approximately 10 µl of hemolymph was used to measure antimicrobial activities. 3-ml logarithmic growth phase cultures of *E. coli* strain 536 or its isogenic deletion mutants in LB were diluted to a final OD_600_ of ~0.002 and growth inhibition was determined using the broth microdilution method in 96-well microtiter plates with an assay volume of 200 µl. Briefly, *G. mellonella* hemolymph (10 μl) was transferred to the microtiter plate containing UPEC strain 536, or the different mutants and incubated overnight at 37°C. Absorbance was measured every 20 minutes at 600 nm to determine bacterial growth inhibition as an indicator of antimicrobial activity. Percentage growth inhibition was calculated by comparing the optical densities of bacterial cultures with and without hemolymph treatment at 1000 minutes. 24 h after inoculation, the bacterial suspensions were serially diluted and plated onto LB agar for CFU determination.

### UPEC survival in the human bladder epithelial cell line RT-112

The human bladder epithelial cell (BEC) line RT-112 (DSMZ No. ACC 418) was cultured in Waymouth’s MB 752/1 medium supplemented with 10% non-heat-inactivated fetal bovine serum (FBS), 1× non-essential amino acids, and 1 mM sodium pyruvate. Cells were maintained at 37°C in a humidified incubator with 5% CO_2_. For infection experiments, RT-112 cells were seeded into 24-well plates at 1.0 × 10^5^ cells/well. Once confluent, cells were incubated overnight in the same complete Waymouth’s medium. The following day, cells were infected with *E. coli* strain 536 or its non-hemolytic mutant 536 HDM at a multiplicity of infection (m.o.i.) of 10 and incubated at 37°C for 2.5 h. To assess the survival of UPEC, extracellular bacteria were killed by gentamicin-containing medium (100 μg/ml) for 1 h, and cells were washed two times with PBS to remove residual antibiotic. Intracellular bacteria were recovered after cell lysis using 0.5 g/L trypsin, w/o EDTA, 0.1% Triton X-100, 0.1% DTT and 0.1 mg/mL DNAse in Dulbecco’s Phosphate-Buffered Saline. Dilutions (1:100) of the lysates were plated on LB agar, and colonies were allowed to grow overnight at 37°C and counted the next day.

### Extraction of RNA, cDNA synthesis and qRT-PCR from *G. mellonella* and human BECs

Five larvae per treatment for each time point were homogenized in 1 ml Trizol reagent (Sigma), and whole animal total RNA was extracted according to the manufacturer’s recommendation. RNA concentrations were determined by spectrophotometry. Complementary DNA was synthesized using the First-strand complementary DNA (cDNA) Synthesis kit (Thermo Fisher Scientific, Schwerte, Germany). The cDNA concentration was determined by spectrophotometry. Quantitative real-time RT-PCR was performed using the CFX 96 real-time PCR system (Bio-Rad Laboratories, Munich, Germany) and SsoAdvanced Universal IT SYBRGreen Smx (Bio-Rad Laboratories, Munich, Germany). For gene expression analysis, total RNA was isolated from infected human BECs, followed by cDNA synthesis and RT-PCR as described above. We used 50 ng of cDNA per reaction to quantify transcripts related to antimicrobial factors and histone acetylation using the primers shown in **Supplementary Table 2**. The amplification parameters comprised an initial activation step at 95°C for 10 min, followed by 39 cycles of denaturation at 95°C for 15s, annealing at 56°C for 15 s and extension at 72°C for 15s. The relative expression levels of the target genes were calculated using the ΔΔCT method, with 18S ribosomal RNA (for *G. mellonella*) and glyceraldehyde-3-phosphate dehydrogenase (for human BECs) used as reference genes for normalization.

### Extraction of histones and measurement of global histone H3K9 and H4K5 acetylation from *G. mellonella* and human BECs

Total histones from larvae after infection with the UPEC strain 536, or the different mutants were extracted using EpiQuik Total Histone Extraction kit (Epigentek, BioCat GmbH, Heidelberg, Germany) according to the manufacturer’s instructions. Briefly, larval homogenates were resuspended in pre-lysis buffer and incubated on ice for 10 min with gentle stirring, followed by centrifugation at 10,000 rpm for 1 min at 4°C. Then, the supernatant containing the cytoplasmic fraction was removed and the cell pellet was resuspended in lysis buffer and incubated on ice for 30 min. Samples were centrifuged at 12,000 rpm for 5 min at 4°C and the supernatant containing acid-soluble proteins was mixed with a balanced buffer solution containing DTT. Histone proteins were quantified using Pierce™ BCA Protein Assay kit (Thermo Scientific), and the extract was aliquoted and stored at −80°C.

Global histone H3K9 and H4K5 acetylation levels in larvae infected with UPEC strain 536 or the different mutants were determined using the EpiQuik global histone H3K9, and H4K5 acetylation assay kits (EpiGentek, BioCat GmbH, Heidelberg, Germany) following the manufacturer’s recommendations. Briefly, the strip wells were coated with histone proteins (200 ng), and acetylated histone H3K9 or H4K5 was detected with a high-affinity antibody. The ratio in infected and uninfected larvae was estimated using a horseradish peroxidase (HRP)-conjugated secondary antibody and the colorimetric signal was quantified by measuring the absorbance at 450 nm. For infected human BECs, extraction of histones and measurement of H4K5 acetylation were performed as described above.

### Data analysis

All experiments were performed a minimum of three times. All figures were created, and statistical analyses were conducted using GraphPad Prism version 8.0.1 for Windows (GraphPad Software, San Diego, CA, USA). Survival curves were plotted using the Kaplan-Meier method. Survival differences were calculated using the log rank test, and P values were adjusted for multiple comparisons using the Bonferroni method. The survival of the individual PAI mutants in the presence of *G. mellonella* hemolymph proteins was statistically analyzed using one-way ANOVA and Tukey’s multiple comparisons test. Gene expression in infected *G. mellonella* larvae was calculated relative to mock-injected larvae (set to 1), and comparisons with UPEC strain 536-infected larvae (control) were evaluated using one-way ANOVA and Dunnett’s multiple comparisons test. Similarly, gene expression in infected RT-112 cells was compared to uninfected controls (set to 1) using one-way ANOVA and Dunnett’s test. Histone acetylation levels were calculated relative to mock-injected larvae (set to 100), and statistical comparisons were made against UPEC strain 536-infected larvae using one-way ANOVA and Dunnett’s multiple comparisons test. Histone acetylation levels in UPEC strain 536-infected bladder epithelial cells (BECs) were compared using unpaired two-tailed Student’s t test. Data distributions were tested for normality using the Shapiro-Wilk test (p < 0.01).

## Results

### Contribution of PAIs to virulence and antimicrobial resistance of UPEC during *G. mellonella* infections

**Figure 2:**
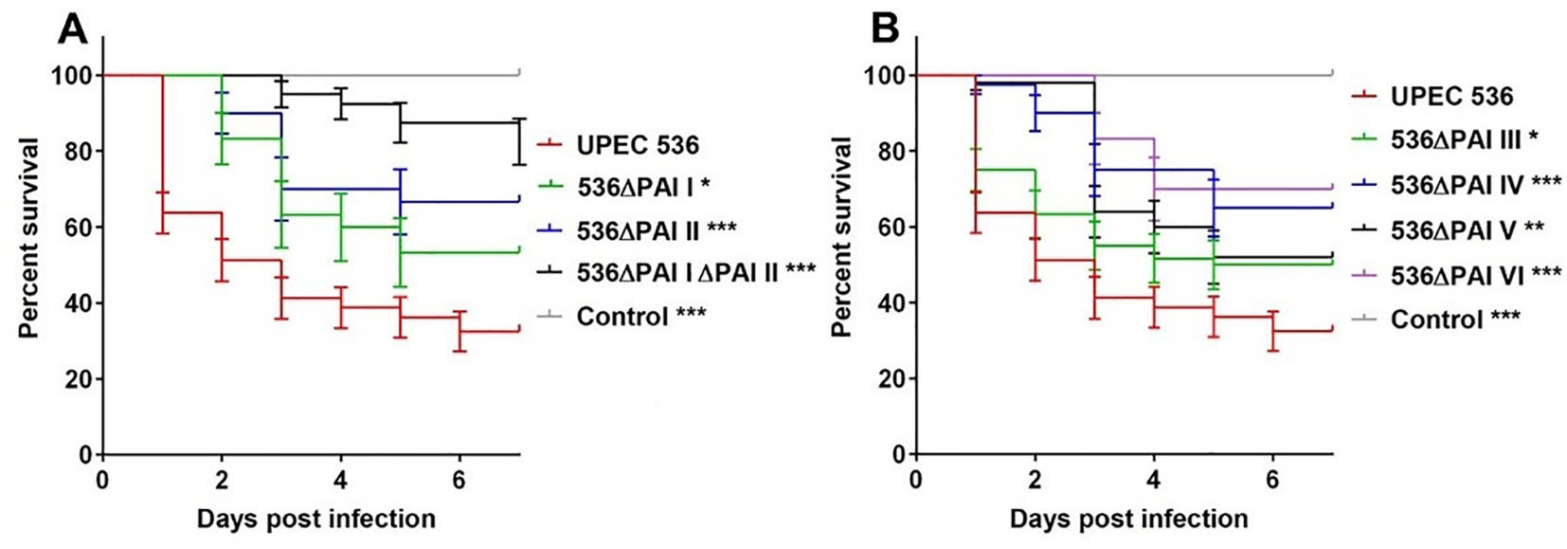
Contributions of PAIs I_536_-PAI VI_536_ to virulence of the *E*. *coli* strain 536 in *G*. *mellonella*. Kaplan-Meier survival plots of larvae after injection with different PAI deletion mutants of UPEC strain 536 showed reduced mortality compared to the control (UPEC strain 536). Deletion of **(A)** PAI I_536_, PAI II_536_, or PAI I_536_ and II_536_ as well as **(B)** PAI III_536_, PAI IV_536_, PAI V_536_ and PAI VI_536_, significantly reduced the killing capacity relative to the control. Larvae injected with an empty needle served as uninfected controls. Results represent the means of at least three independent determinations for 10 animals per treatment (*P < 0.05; **P < 0.005; ***P < 0.0005).

To investigate the contribution of PAIs I_536_ – VI_536_ to UPEC virulence we infected *G. mellonella* larvae with individual PAI deletion mutants (*E. coli* strain 536ΔPAI I – *E. coli* strain 536ΔPAI VI) by injection of 1.0 × 10^4^ CFU per larva and monitored mortality for up to 7 days post-injection at 37°C. No deaths were recorded when larvae were injected with 0.9% NaCl (negative control). We observed significant differences in the mortality rates of larvae infected with the different PAI mutants in comparison to the UPEC strain 536 at a dose of 1.0 × 10^4^ CFU per larva (P<0.0001). Injection of the UPEC strain 536 resulted in maximum mortality of larvae (survival rate 32%). However, the mortality rates were reduced following the deletion of individual PAIs with the strongest virulence attenuation observed by *E. coli* strain 536ΔPAI VI (survival rate 70%) followed by *E. coli* strain 536ΔPAI II (survival rate 66%), *E. coli* strain 536ΔPAI IV (survival rate 65%), *E. coli* strain 536ΔPAI I (survival rate 53%), *E. coli* strain 536ΔPAI V (survival rate 52%), and *E. coli* strain 536ΔPAI III (survival rate 50%) (**Figures 2A, B**). The loss of PAIs appeared to have a cumulative effect, as the simultaneous deletion of PAI I_536_ and PAI II_536_ (*E. coli* strain 536ΔPAI I ΔPAI II) further attenuated virulence in larvae (survival rate 82%) (**Figure 2A**). The virulence attenuation of the *E. coli* strain 536ΔPAI I ΔPAI II mutant was not only evident in comparison to the UPEC strain 536 but also to most individual PAI deletion mutants (P<0.0001). Virulence attenuation was also significantly different between mutants lacking PAI III_536_ and PAI VI_536_ (P<0.05) and between the PAI II_536_ and PAI III_536_ deletion mutants (P<0.05).

**Figure 3:**
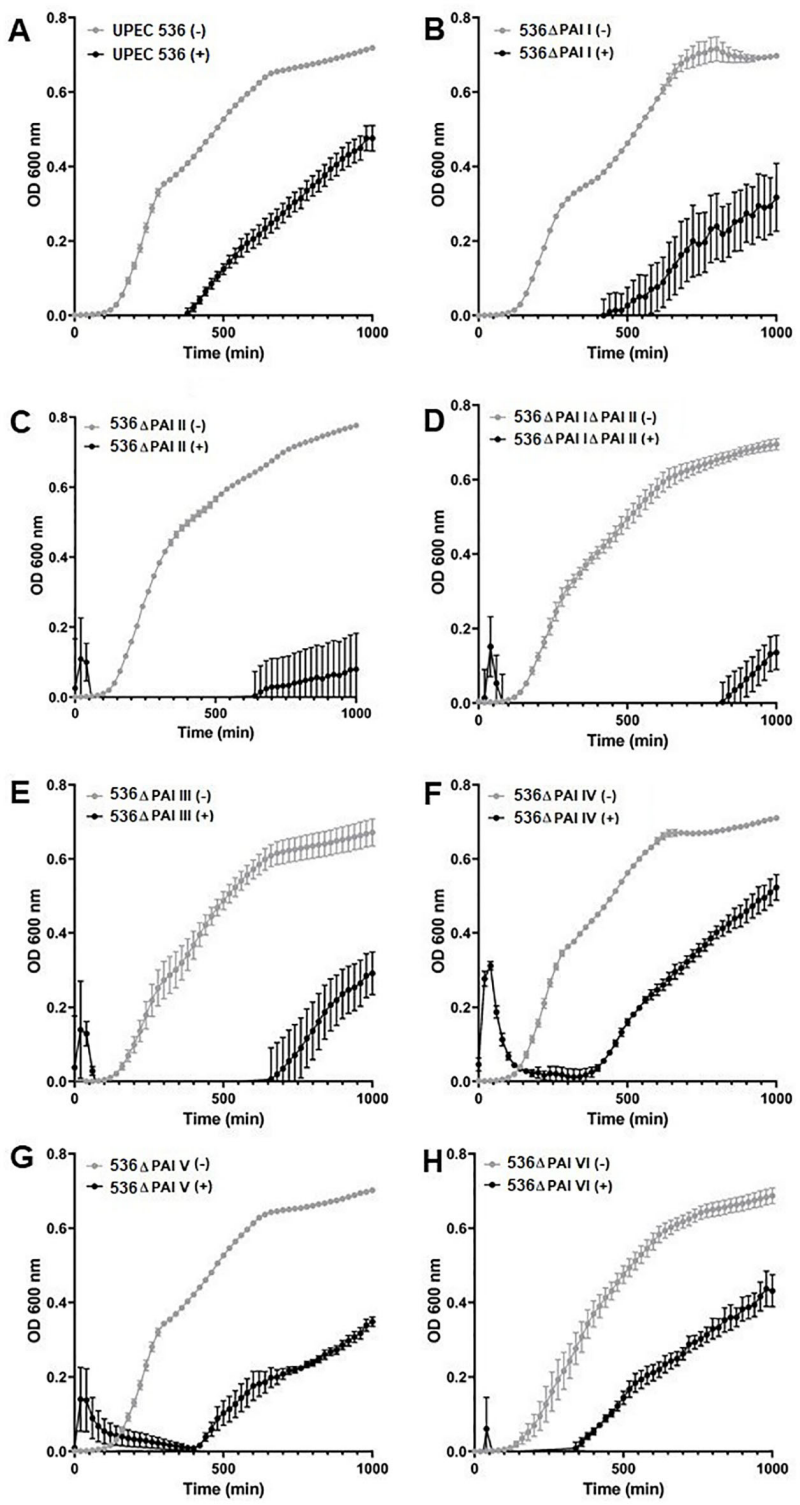
Contribution of PAls I_536_-VI_536_ to *E. coli* strain 536 growth in *G. mellonella* hemolymph. Growth of the **(A)** wild-type UPEC strain 536 or the deletion mutants of **(B)** PAI I_536_, **(C)** PAI II_536_, **(D)** PAI I_536_ and PAI II_536_, **(E)** PAI III_536_, **(F)** PAI IV_536_, **(G)** PAI V_536_, or **(H)** PAI VI_536_ in the presence of the pre-immune activated *G. mellonella* hemolymph. Bacteria were cultured in LB supplemented with 10 µL hemolymph, and the optical density at 600 nm (0D600) was measured every 20 minutes. +/— refers to bacteria grown in the presence (+) or absence (—) of *G. mellonella* hemolymph. Results represent mean values of at least three independent determinations ± SE.

**Figure 4:**
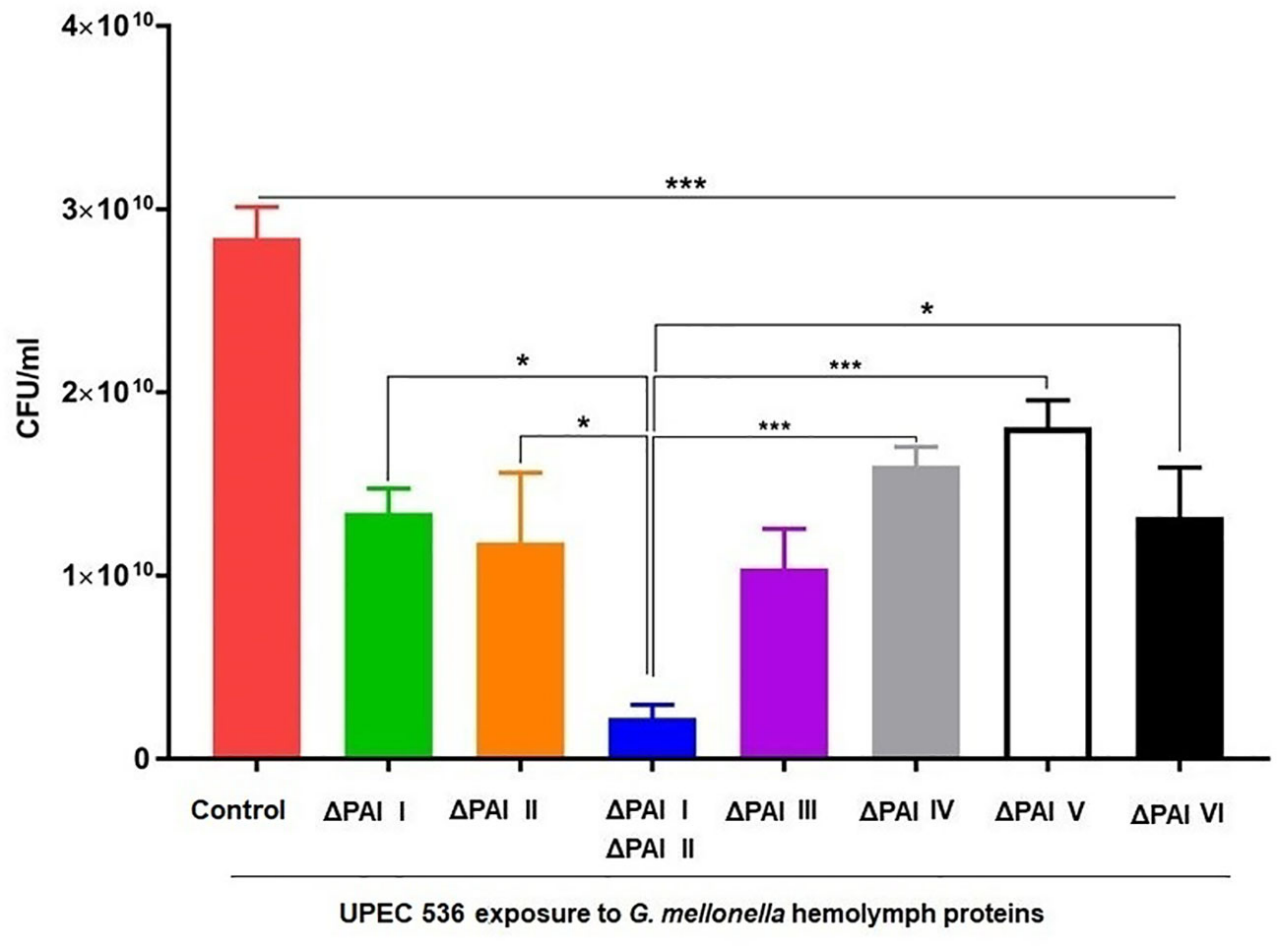
Contribution of PAIs I_536_-VI_536_ to *E. coli* strain 536 survival in antimicrobial *G. mellonella* hemolymph. The wild-type UPEC strain 536 (control) or the PAI deletion mutants were cultured in LB supplemented with 10 μl *G. mellonella* hemolymph from pre-immune activated larvae. The bacterial cultures after 24 h were serially diluted and grown on LB agar for the estimation of colony-forming units (CFUs). Results represent mean values of at least three independent determinations ± SE. (*P < 0.05; ***P < 0.0005).

We investigated whether the reduced mortality of larvae caused by different PAI mutants is influenced by their varying sensitivity to antimicrobial proteins, which are key components of insect innate immunity (**Figures 3**, **4**). For this, hemolymph samples were isolated from larvae following injection with heat-killed *E. coli* DH5α eliciting antimicrobial innate immune response. PAI mutants of *E. coli* strain 536 grown to the logarithmic phase were then incubated in 10 µl pre-immune activated hemolymph at 37°C for over 16 h (**Figures 3A–H**). The UPEC strain 536 (**Figure 3A**) showed low sensitivity (growth reduction 33%) to hemolymph compared to the untreated control, whereas the *E. coli* strain 536ΔPAI I ΔPAI II mutant (**Figure 3D**) exhibited one of the highest sensitivities (growth reduction 80%) in LB (**Figure 4**, **Supplementary Table 3**). Similarly, the *E. coli* strains 536ΔPAI I (**Figure 3B**) (growth reduction 54%), 536ΔPAI II (**Figure 3C**) (growth reduction 83%), 536ΔPAI III (**Figure 3E**) (growth reduction 56%), and 536ΔPAI V (**Figure 3G**) (growth reduction 50%) were also strongly sensitive to the antimicrobial hemolymph. In contrast, the *E. coli* strain 536ΔPAI IV (**Figure 3F**) (growth reduction 26%) and 536ΔPAI VI (**Figure 3H**) (growth reduction 36%) only displayed reduced sensitivity (**Supplementary Table 3**). Besides growth inhibition, UPEC survival was reduced significantly following individual deletion of PAIs in *G. mellonella* hemolymph. In addition to the analysis of bacterial growth in hemolymph via optical density (**Figure 3**), we also measured bacterial colony-forming units in hemolymph. This experiment demonstrated that although UPEC survival was generally reduced in all PAI mutants relative to the UPEC strain 536, maximum reduction in UPEC survival was observed for the *E. coli* strain 536ΔPAI I ΔPAI II (**Figure 4**). Accordingly, the deletion of PAI IV_536_ and PAI VI_536_ showed different effects on the mortality of the infected larvae and on the sensitivity of the infecting *E. coli* mutants to antimicrobial hemolymph. In this respect, a strong reduction in bacterial pathogenicity did not always correlate with an increased sensitivity to antimicrobial proteins in the hemolymph of *G. mellonella* larvae.

### Impact of PAIs on the transcriptional reprogramming of innate immunity in UPEC-infected *G. mellonella*

**Figure 5:**
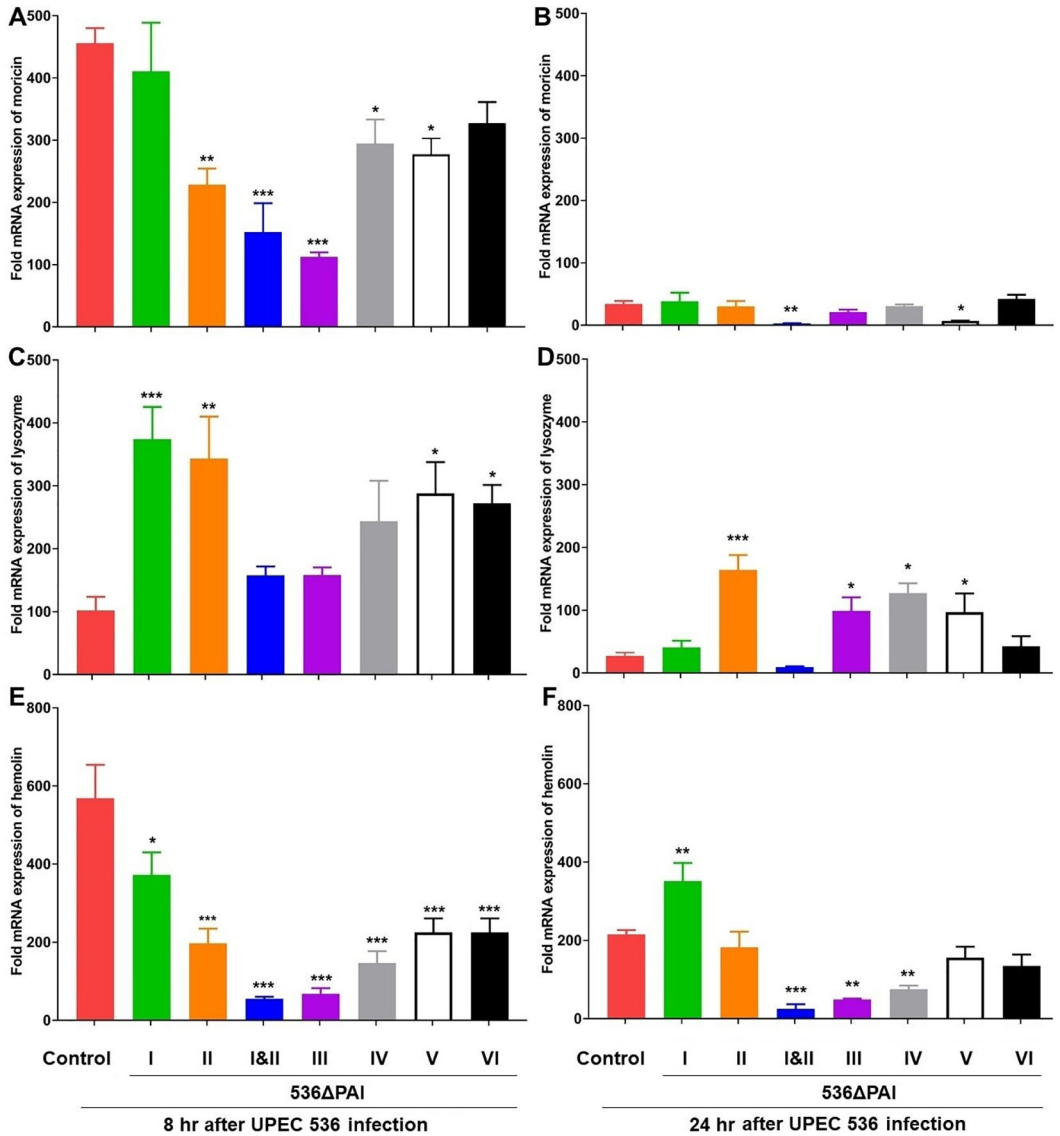
Impact of PAIs I_536_-VI_536_ on the transcriptional activation of antimicrobial gene expression in *E. coli* strain 536-infected *G. mellonella*. The expression of **(A, B)** moricin, **(C, D)** lysozyme, **(E, F)** hemolin genes was assessed in larvae at 8 h **(A, C, E)** and 24 h **(B, D, F)** post-injection with different PAI deletion mutants (536ΔPAI I - 536ΔPAI VI and 536ΔPAI I ΔPAI II) of UPEC strain 536 by quantitative real-time RT-PCR. Basal expression in the infected larvae was calculated as fold-change relative to mock-injected control larvae and normalized to the 18S rRNA housekeeping gene. Statistical differences in gene expression were calculated relative to larvae infected with the UPEC strain 536 (control). Results represent mean values of at least three independent determinations ± SE (*P < 0.05; **P < 0.005; ***P < 0.0005).

We investigated the impact of PAIs on the expression of antimicrobial peptide (AMP)- and other antimicrobial factor-encoding genes in larvae at 8- and 24 h post-infection using RT-PCR. We observed strong upregulation of genes encoding antimicrobial factors e.g., moricin (**Figures 5A, B**), lysozyme (**Figures 5C, D**), hemolin (**Figures 5E, F**), IMPI (**Supplementary Figures 1A, B**), and galiomicin (**Supplementary Figures 1C, D**) throughout the infection period with the individual PAI mutants (*E. coli* strain 536ΔPAI I - *E. coli* strain 536ΔPAI VI), the *E. coli* strain 536ΔPAI I ΔPAI II, or the UPEC strain 536 compared to uninfected larvae (**Figure 5**, **Supplementary Figure 1**). Although AMP expression was high at 8 h, it was reduced by 24 h. In contrast, other innate immunity-related genes such as apolipophoricin-III (**Supplementary Figures 2A, B**) and prophenoloxidase (**Supplementary Figures 2C, D**), exhibited weak expression following infection.

The amphipathic α-helical AMP moricin exhibits activity against both Gram-negative and Gram-positive bacteria (27). We found that the reduction in moricin expression at 8 h following infection with PAI II_536_, PAI III_536_, PAI IV_536_, and PAI VI_536_ deletion mutants was restored by 24 h, resembling the response seen with the UPEC strain 536 (**Figures 5A, B**). However, its expression was reduced after infection with the *E. coli* strain 536ΔPAI I ΔPAI II and *E. coli* strain 536ΔPAI V at both 8 and 24 hours. Next, we tested the expression of lysozyme as it exhibits activity against Gram-positive and Gram-negative bacteria (28). Induction in lysozyme expression at 8 h following infection with PAI I_536_ and PAI VI_536_ deletion mutants was restored by 24 h, resembling the response seen with the UPEC strain 536 (**Figures 5C, D**). However, its expression remained elevated after infection with the *E. coli* strains 536ΔPAI II, 536ΔPAI IV and 536ΔPAI V at both 8 h and 24 h (**Figures 5C, D**). In addition, we analyzed the contribution of the PAIs to the expression of the insect metalloprotease inhibitor (IMPI) and the defensin galiomicin (29, 30). IMPI was upregulated in infected larvae by the *E. coli* strain 536ΔPAI I and *E. coli* strain 536ΔPAI V relative to the UPEC strain 536–8 h post-infection. However, the expression of these genes was downregulated upon infection *E. coli* strain 536ΔPAI I ΔPAI II 24 h post-infection (**Supplementary Figures 1A, B**). Galiomicin was induced in response to UPEC infection, and we observed relative upregulation of this gene by the PAI mutant strains 536ΔPAI I, 536ΔPAI V and 536ΔPAI VI 8 h and 24 h post-infection (**Supplementary Figures 1C, D**). No changes in galiomicin expression were observed in larvae infected with the *E. coli* strains 536ΔPAI II, 536ΔPAI I ΔPAI II, 536ΔPAI III, or 536ΔPAI IV relative to the UPEC strain 536 at both 8 h and 24 h post-infection.

Genes coding for other innate immune-related factors, such as hemolin, apolipophoricin-III and prophenoloxidase, exhibited different expression patterns than the AMP genes. Hemolin, a member of the immunoglobulin (Ig) superfamily, functions as a pathogen-recognition molecule in the defense against infection (31). We observed a less pronounced upregulation of the hemolin-encoding gene in larvae infected with the PAI mutants relative to the UPEC strain 536–8 h post-infection (**Figure 5E**). After 24 h of infection, the upregulation of the hemolin gene was weaker by a factor of 3 when infected with the UPEC strain 536 than after 8 h of infection, whereas the expression level of the hemolin-encoding gene did not change significantly between 8 h and 24 h post-infection when infected by the different PAI mutants (**Figure 5F**). Apolipophoricin-III (apoLp-III), an insect equivalent of human apolipoprotein E, is a lipid-binding protein that plays a vital role in lipid transport and immune defense in insects (32). We found that the expression of this gene was either downregulated or transiently expressed compared to the uninfected larvae in response to infection with the UPEC strain 536 or different PAI mutants after 8 h and 24 h of infection. The expression of the prophenoloxidase-encoding gene required for melanization in insects was reduced by the UPEC strain 536 and the PAI mutants after 8 h (**Supplementary Figure 2C**). After 24 h of infection, however, prophenoloxidase expression was significantly increased by *E. coli* strains 536ΔPAI I ΔPAI II, 536ΔPAI III, and 536ΔPAI V (**Supplementary Figure 2D**). This suggests that the innate immune response of the infected larvae reacts individually to different PAI mutants during the infection.

### Impact of PAIs on the expression of HDACs and HATs in *G. mellonella* infected with UPEC

**Figure 6:**
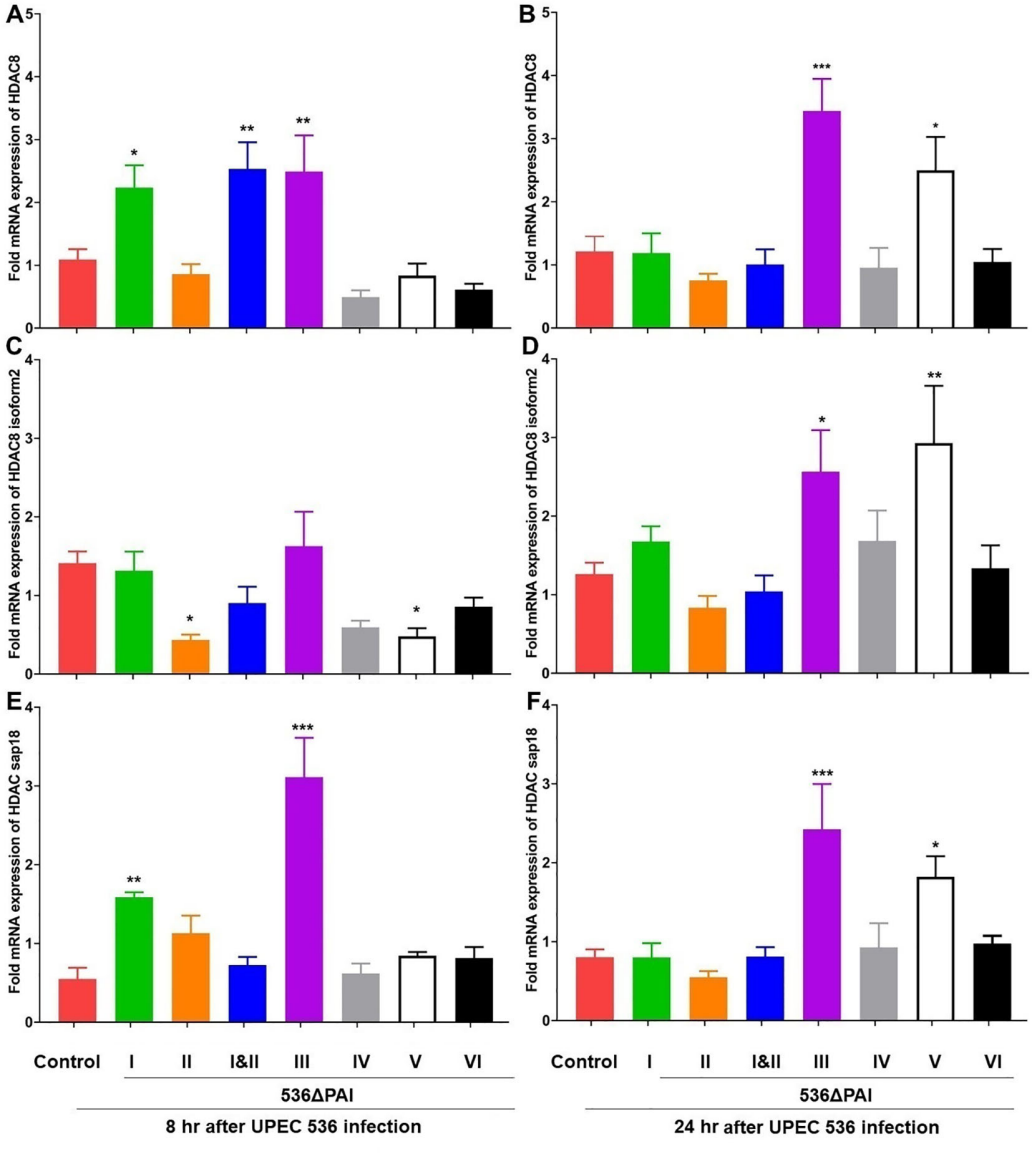
Impact of PAIs I_536_-VI_536_ on the transcriptional activation of histone deactylase (HDAC) gene expression in *E. coli* strain 536-infected *G. mellonella*. The expression of **(A, B)** HDAC8, **(C, D)** HDAC8 isoform2, **(E, F)** HDAC Sap18 genes was assessed in larvae at 8 h **(A, C, E)** and 24 h **(B, D, F)** post-injection with different PAI deletion mutants (536ΔPAI I - 536ΔPAI VI and 536ΔPAI I ΔPAI II) of UPEC strain 536 by quantitative real-time RT-PCR. Basal expression in infected larvae was calculated as fold-change relative to mock-injected control larvae and normalized to the 18S rRNA housekeeping gene. Statistical differences in gene expression were calculated relative to larvae infected with the UPEC strain 536 (control). Results represent mean values of at least three independent determinations ± SE (*, P < 0.05; **, P < 0.005; ***, P < 0.0005).

We investigated the expression of histone deacetylases (HDACs) and histone acetyltransferases (HATs) and found that their expression was influenced by the specific PAIs of *E. coli* strain 536–8 h and 24 h after infection. Infection with *E. coli* strains 536ΔPAI I, 536ΔPAI III, 536ΔPAI V or 536ΔPAI I ΔPAI II resulted in upregulation of selected HDAC and HAT genes. Specifically, the expression of HDAC8 and HDAC Sap18 was upregulated in larvae 8 h post-infection with the *E. coli* strains 536ΔPAI I, 536ΔPAI I ΔPAI II and 536ΔPAI III (**Figures 6A–F**), with this increased expression persisting at 24 h only in larvae challenged with the *E. coli* strain 536ΔPAI III. The expression of HDACs in the remaining PAI mutant-infected larvae was similar to that of infection with the UPEC strain 536, except for the HDAC8 isoform2 subunit, which was downregulated by the PAI II_536_ and PAI V_536_ mutants 8 h post-infection (**Figure 6C**). PAI V_536_ mutants exhibited increased expression of HDAC genes at 24 h post-infection.

**Figure 7:**
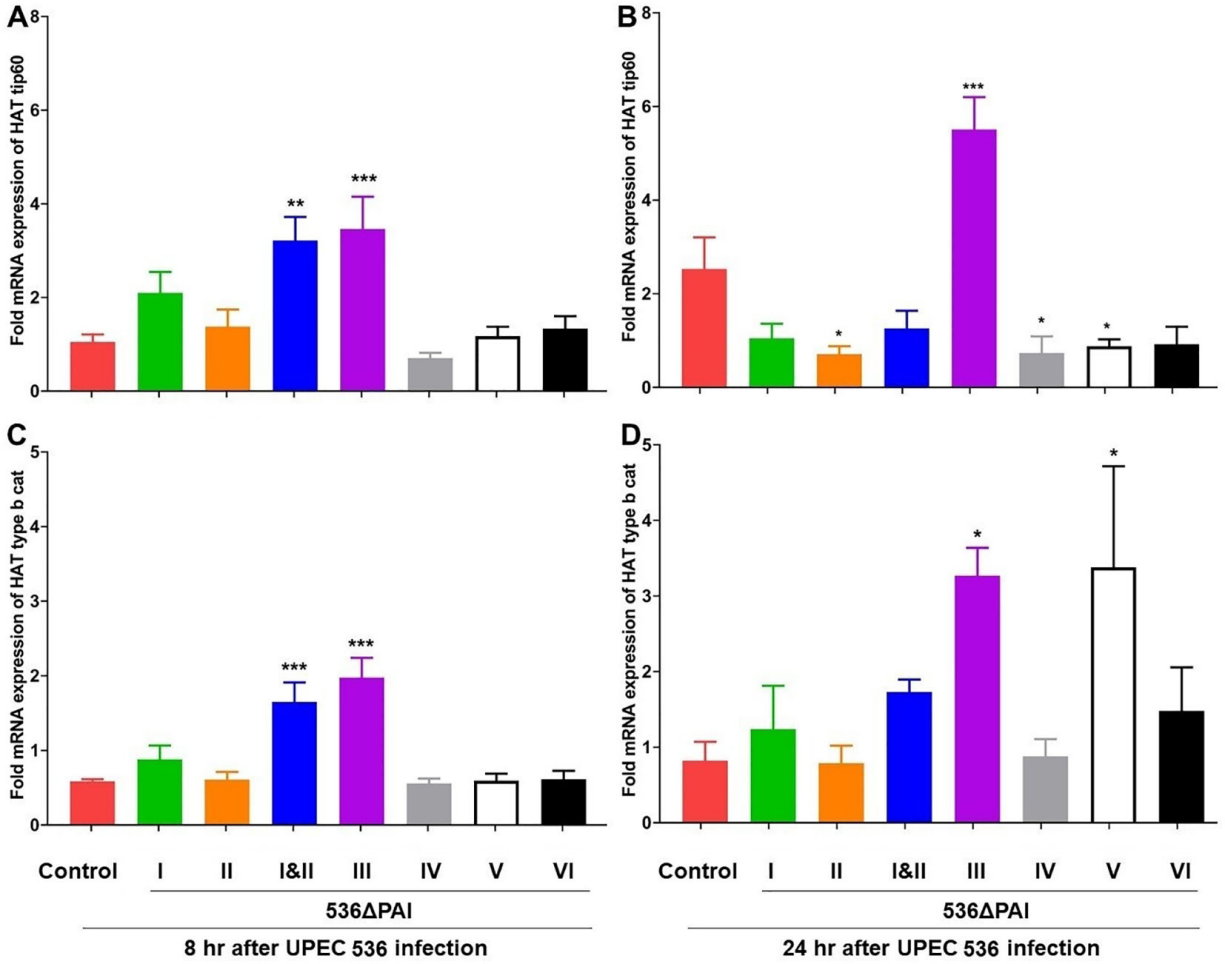
Impact of PAIs I_536_-VI_536_ on the transcriptional activation of histone acetyltransferase (HAT) gene expression in *E. coli* strain 536-infected *G. mellonella*. The expression of **(A, B)** HAT Tip60, and **(C, D)** HAT type b cat genes was assessed in larvae at 8 h **(A, C)** and 24 h **(B, D)** post-injection with different PAI deletion mutants (536ΔPAI I – 536ΔPAI VI and 536ΔPAI I ΔPAI II) of UPEC strain 536 by quantitative real-time RT-PCR. Basal expression in infected larvae was calculated as fold-change relative to mock-injected control larvae and normalized to the 18S rRNA housekeeping gene. Statistical differences in gene expression were calculated relative to larvae infected with the UPEC strain 536 (control). Results represent mean values of at least three independent determinations ± SE (*P < 0.05; **P < 0.005; ***P < 0.0005).

Similarly, the expression of HAT genes (HAT tip60 and HAT type B catalytic) was upregulated 8 h post-infection with the *E. coli* strains 536ΔPAI I ΔPAI II and 536ΔPAI III (**Figures 7A–D**). After 24 h of infection, the expression of these two HAT genes was upregulated by the *E. coli* strains 536ΔPAI III and 536ΔPAI V. HAT gene expression in larvae infected by the other PAI deletion mutants was unaffected or slightly reduced relative to that of the UPEC strain 536 at both 8 h and 24 h post-infection. From these results, it can be concluded that the expression of enzymes contributing to chromatin dynamics also reacts depending on the presence of individual PAIs.

### PAI-mediated epigenetic regulation of histone acetylation in UPEC-infected *G. mellonella*

**Figure 8:**
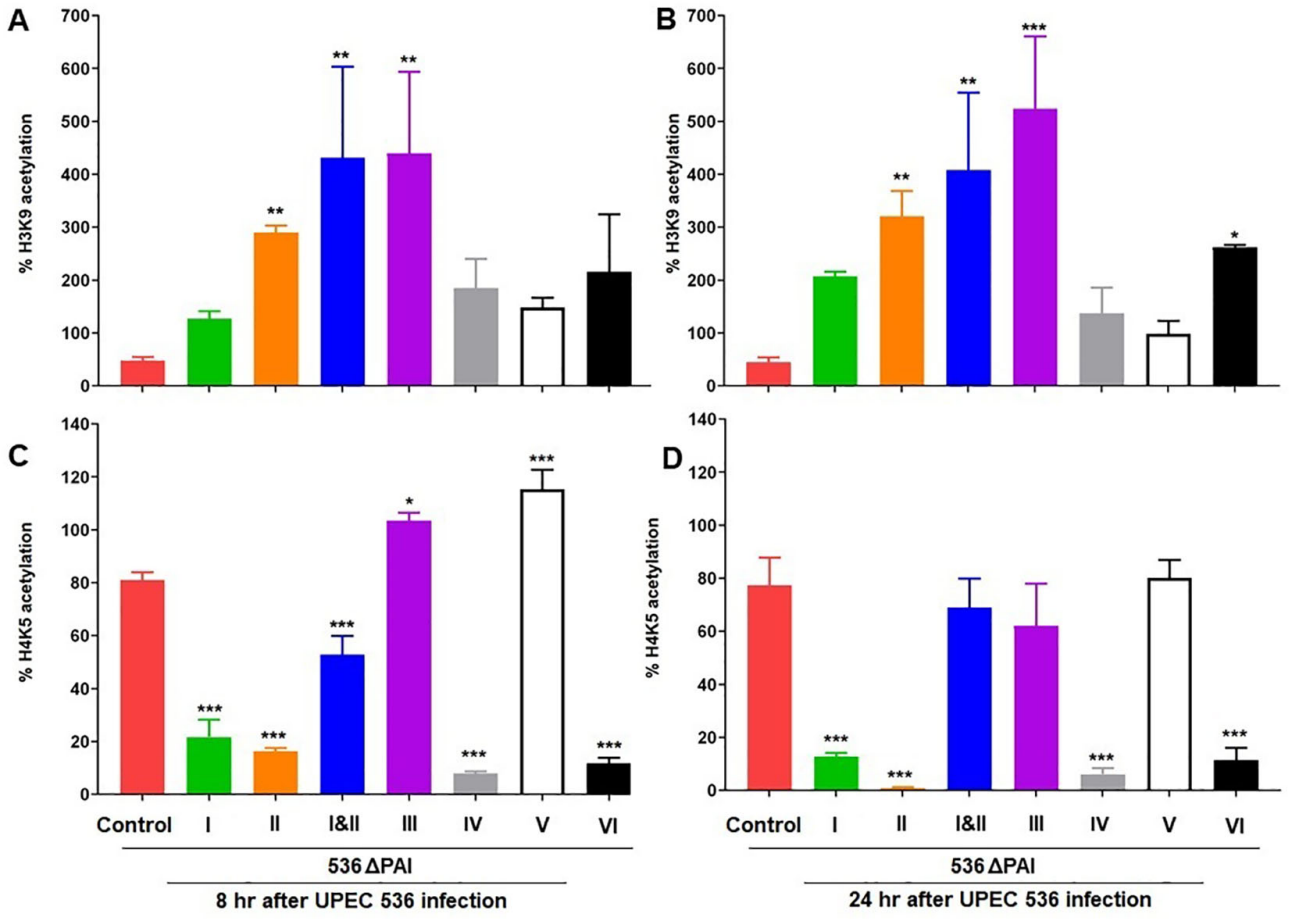
Contribution of PAIs I_536_-VI_536_ of the *E. coli* strain 536 in altering histone acetylation in infected *G. mellonella* larvae. Changes in H3K9 **(A, B)**, and H4K5 **(C, D)** acetylation levels was assessed in larvae at 8 h **(A, C)** and 24 h **(B, D)** post-injection with different PAI deletion mutants (536ΔPAI I - 536ΔPAI VI and 536ΔPAI I ΔPAI II) of UPEC strain 536 by ELISA. Statistical differences in gene expression were calculated relative to larvae infected with the UPEC strain 536 (control). Results represent mean values of at least three independent determinations ± SE (*P < 0.05; **P < 0.005; ***P < 0.0005).

The expression of HDAC and HAT genes can regulate the acetylation of specific lysine residues in core histones. We complemented our HDAC-HAT gene expression analysis by characterizing the acetylation status of specific histone marks in larvae infected with the UPEC strain 536 or individual PAI mutants. Histones were isolated from whole larvae infected with the UPEC strain 536 or the PAI mutants (*E. coli* strain 536ΔPAI I - *E. coli* strain 536ΔPAI VI) for the antibody-based detection of global H3K9 and H4K5 acetylation (**Figures 8A, B**). We selected H3K9 and H4K5 for this analysis as these histone markers were differentially regulated by UPEC and commensal-like asymptomatic bladder colonizing *E. coli* strains in *G. mellonella*, in addition to their role in regulating immune responses in mammals (23). Overall, H3K9 and H4K5 acetylation were reduced by infection with the UPEC strain 536 compared to uninfected control larvae (H3K9 and H4K5 acetylation levels in control larvae shown as 100%). However, histone acetylation levels varied depending on infection with the different PAI mutants, the histone modifications (H3K9 or H4K5), and the infection time point (8 h or 24 h).

We observed significant differences in H3K9 and H4K5 acetylation in larvae infected with selected PAI mutants. Infection with most PAI mutants led to increased H3K9 acetylation, while H4K5 acetylation levels were reduced by several PAI mutants compared to the UPEC strain 536. Notably, H3K9 acetylation increased significantly upon infection with *E. coli* strains 536ΔPAI II, 536ΔPAI I ΔPAI II, and 536ΔPAI III at both 8 h and 24 h post-infection, a trend that was also observed by the *E. coli* strain 536ΔPAI VI at 24 h post-infection. Conversely, H4K5 acetylation was reduced upon infection with most PAI mutants except *E. coli* strain 536ΔPAI III and *E. coli* strain 536ΔPAI V after 8 h of infection. Our results demonstrate that specific PAIs of UPEC play a key role in modulating HDAC-HAT expression, thereby influencing the acetylation of distinct histone marks in infected larvae.

### Validation of PAI-encoded virulence factor-driven histone acetylation changes in *G. mellonella* during infection with human bladder epithelial cells

**Figure 9:**
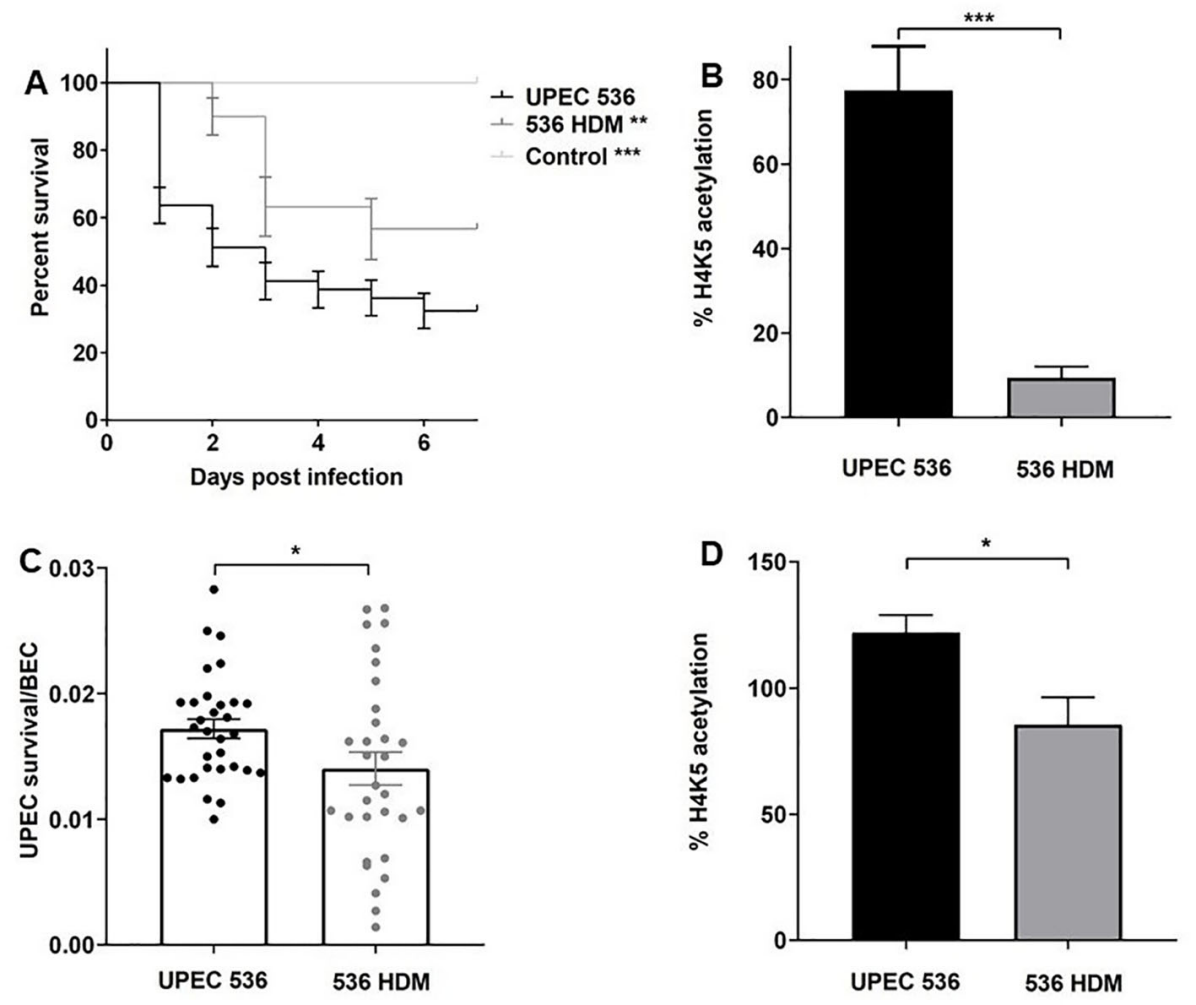
Contributions of α-hemolysin to virulence of the *E. coli* strain 536 in *G. mellonella* and human RT-112 bladder epithelial cells. The impact of α-hemolysin on host-pathogen interactions was assessed by comparing the UPEC strain 536 with *E. coli* strain 536 HDM. **(A)** Kaplan—Meier survival curves show reduced mortality in *G. mellonella* larvae infected with the non-hemolytic mutant strain. **(C)** Similarly, intracellular survival of UPEC in RT-112 cells was significantly lower for the mutant compared to the wild-type. **(B, D)** H4K5 acetylation levels were measured by ELISA 24 h post-injection in larvae and 2.5 h post-infection in RT-112 cells. Statistical differences were calculated relative to infection with the UPEC strain 536 (control). Results represent mean values of at least three independent determinations ± SE (*, P < 0.05; **, P < 0.005; ***, P < 0.0005).

To assess whether host response changes induced by PAI-encoded virulence factors in larvae are conserved in humans, we infected *G. mellonella* larvae and human RT-112 BECs with the *E. coli* strain 536 and its non-hemolytic mutant 536 HDM (**Figures 9A–D**). Deletion of both hemolysin (*hly*I and *hly*II) gene clusters led to virulence attenuation in larvae and reduced UPEC survival in RT-112 cells compared to the UPEC strain 536 (**Figures 9A, C**). We then focused on H4K5 acetylation to determine whether the absence of α-hemolysin causes similar epigenetic effects in both model systems. Our results show that infection with the *E. coli* strain 536 HDM led to a decrease in H4K5 acetylation in both larvae and RT-112 cells relative to UPEC strain 536 infection (**Figures 9B, D**). These findings indicate that PAI-driven host responses, such as changes in H4K5 acetylation, are consistent between larvae and human BECs, supporting the use of *G. mellonella* as a suitable model for studying specific aspects of UPEC pathogenesis in humans.

## Discussion

Fundamental principles of differentiation between pathogenic and commensal bacteria by eukaryotic cells and the associated regulation of the innate immune response have been intensively studied for many years. At the molecular level, much is already known about the specific recognition of pathogen-associated molecular patterns (PAMPs) by Toll-like (TLR) and NOD-like receptors (NLRs), thereby inducing signaling pathways that lead to the activation of an inflammatory response (33, 34).

UPEC can manipulate the innate immunity of the host by expressing virulence factors located at distinct PAIs. One way this is achieved is through direct interactions between virulence factors and specific host proteins, constituting different immune signaling mechanisms (6). Alternatively, the asymptomatic bladder colonizer *E. coli* 83972 can counteract destructive inflammatory responses in the bladder by modulating the function of the RNA polymerase II (35). Additionally, regulation of gene expression can also occur at the epigenetic level involving differential modification of histones (11). It has been shown that UPEC can regulate histone acetylation in infected host cells (16). However, the exact role of specific virulence factors in this process is currently unknown. We have shown that discriminatory host responses to infection by UPEC or the ABU *E. coli* isolate 83972 in *G. mellonella* larvae also involve changes at the epigenetic level (23, 26). Here we studied the contribution of individual PAIs to UPEC infection in *G. mellonella* larvae with a particular focus on chromatin dynamics and the regulation of the innate host response. This study extends and complements similar approaches to systematically assess the contribution of individual PAIs to the pathogenicity of UPEC strain 536 in different surrogate infection models, such as mouse, *Caenorhabditis elegans*, and *Dictyostelium discoideum* (17, 36, 37). The contribution of PAI I_536_ - PAI V_536_ to the pathogenicity of *E. coli* strain 536 was analyzed in a murine model of ascending UTI. The deletion of PAI I_536_, II_536_, or III_536_ led to a similar increase in murine survival. The impact of PAI loss appeared to be cumulative, as evidenced by the further attenuation upon simultaneous deletion of PAI I_536_ and II_536_. This observation could not be fully explained by the loss of α-hemolysin determinants, as a less pronounced decrease in pathogenicity was reported in an isogenic double *hly* mutant in the same experimental setup. These results indicated that other PAI I_536_ and II_536_-encoded factors influence UPEC pathogenicity, too. Conversely, in a murine sepsis model, the deletion of individual PAIs did not have a substantial impact on the pathogenicity of UPEC 536. However, the simultaneous deletion of PAI I_536_ and II_536_ resulted in a significant increase in survival rates among infected mice. In the septicemia model, a similar attenuation was observed in mice infected with isogenic *hly* mutants, indicating that, in contrast to the ascending UTI model, an individual virulence factor, α-hemolysin, plays a predominant role in bacteremia (17). In another murine septicemia model, it has been demonstrated that the PAIs of UPEC strain 536 contribute differentially to bacterial pathogenicity, exhibiting an additive effect in this infection model (38). Furthermore, data from this murine septicemia model demonstrate that the effect of a specific PAI, i.e., PAI IV_536_, on pathogenicity is contingent upon the host strain background and genome content (39). However, the impact of individual PAIs on epigenetic changes in the infected host remains to be elucidated.

We therefore infected *G. mellonella* larvae with single deletion mutants of PAI I_536_ to PAI VI_536_ as well as a PAI I_536_ and PAI II_536_ double mutant of UPEC strain 536 to analyze specific epigenetic changes at the level of histone acetylation. The joint loss of PAI I_536_ and PAI II_536_ not only led to the highest level of virulence attenuation compared to the individual PAI mutants in *G. mellonella* (**Figure 2**), but also severely impaired the growth and survival of the UPEC strain in the presence of antimicrobial hemolymph proteins (**Figure 3**). Among the individual PAI deletion mutants, *E. coli* strain 536ΔPAI II was more sensitive to *G. mellonella* hemolymph than UPEC strain 536 or the other PAI deletion mutants studied (**Figure 3**). Our understanding of the contribution of PAIs to antimicrobial resistance is limited, despite evidence indicating their importance in UPEC resistance to multiple antibiotics, human serum and lysozyme (40, 41). As already described above, our results from the *G. mellonella* model are in accordance with a murine urosepsis model, where both PAI I_536_ and PAI II_536_ were essential for pathogenesis (17). In the murine urosepsis model, it was not possible to distinguish the effects of individual PAIs based on survival rates. However, differences in their LD50 values underscore variations in their ability to interact with and evade the host immune response that we observed in *G. mellonella* larvae (**Figures 2**, **3**). In the *G. mellonella* model, the contribution of individual PAIs to the pathogenic potential of UPEC strain 536 could be distinguished. Notably, although only PAI III_536_, PAI I_536_, and PAI II_536_ have been shown to play major roles in UPEC virulence in other infection models, their virulence profiles are consistent with our comprehensive analysis of all PAI deletion mutants of *E. coli* 536 in the *G. mellonella* model. This confirms that also in the insect larval infection model the virulence of individual PAI mutants can be studied reproducibly and in agreement with other infection models (17, 36, 37). However, the increased sensitivity of individual PAI mutants to the hemolymph of the larvae did not always correlate with increased larval survival, as observed in infections with the *E. coli* strains 536ΔPAI IV and 536ΔPAI VI, suggesting increased vulnerability to alternative immune defense mechanisms (**Figures 2**–**4**). This observation highlights that loss of selected PAIs (PAI IV_536_ and PAI VI_536_) can impose significant biological costs for maintaining both high virulence and strong antimicrobial resistance, resulting in trade-offs that impact bacterial fitness. A comparable situation was observed with *Staphylococcus aureus*, where daptomycin resistance led to attenuated virulence in the fruit fly (*Drosophila melanogaster*) model, an effect attributed to increased sensitivity to the host phenoloxidase system rather than to AMPs (42). Accordingly, we demonstrate here that individual PAIs differentially influence host responses, namely epigenetic mechanisms of histone acetylation/deacetylation and antimicrobial gene expression, in *G. mellonella*, thus highlighting their distinct roles in UPEC pathogenesis.

The life span of *G. mellonella* larvae was extended by inhibiting HDACs despite the associated severe tissue damage and septic shock (43). In our study, histone acetylation in *G. mellonella* showed both qualitative differences (in the spectrum of histone markers) and quantitative variations (in the expression levels of HAT/HDAC-encoding genes and the abundance of the corresponding modified histones) following infection with selected PAI mutants. As with the UPEC strain CFT073, also infection with the UPEC strain 536 affected HDAC and HAT gene transcription (**Figure 6**). We observed changes in HDAC and HAT gene expression, comparable to larvae, in human RT-112 BECs as well as in bladder and kidney tissue of mice that were intravesically infected with the *E. coli* strain 536 (**Supplementary Methods**, **Supplementary Figures 3**, **4**). In the human RT-112 cell line, UPEC infection led to the upregulation of HDAC genes (HDAC2, HDAC4, HDAC9) and HAT genes (KAT6A, KAT6B, KAT7). Tissue-specific alterations were also observed in a mouse model of UPEC infection, with modest upregulation of the HDAC gene SIRT3 in the kidney and the HAT gene KAT2A in the urinary bladder 24 h post-infection (**Supplementary Figure 4**). This pattern of HDAC and HAT expression in mammalian cells mirrors the responses we have observed in *G. mellonella*. We are aware that the importance of various factors such as infection dynamics, tissue type, UPEC localization in the infected organ, severity of infection, differential expression of different HDAC and HAT genes, as well as sufficient sample size are also required to achieve a consolidated and improved understanding of HDAC-HAT expression in infected mammalian hosts. Additionally, it remains to be determined whether the tissue-specific changes in HDAC and HAT expression seen in the murine model of ascending urinary tract infection are associated with the reduced acetylation of H3K9 and H4K5, as observed in UPEC-infected *G. mellonella* larvae compared to uninfected controls (**Figure 8**).

The abundance of the histone markers in larvae infected with the UPEC strain 536 was either reversed or transiently regulated after infection with the selected PAI mutants. We observed a contrasting epigenetic response by selected PAI mutants in larvae. *E. coli* strains 536ΔPAI III and 536ΔPAI V caused an increase in transcript levels of HDAC/HAT-encoding genes as well as significantly increased H3K9 or H4K5 acetylation (**Figures 6**–**8**), whereas infection with the *E. coli* strains 536ΔPAI I, 536ΔPAI II, 536ΔPAI IV and 536ΔPAI VI resulted in significant downregulation of H4K5 acetylation levels (**Figure 8**). Interestingly, simultaneous deletion of PAI I_536_ and PAI II_536_ (*E. coli* strain 536ΔPAI I ΔPAI II) led to elevated expression of selected HDAC and HAT genes, along with trend wise increased H3K9 and H4K5 acetylation in larvae, compared to infection with either of the single mutants (*E. coli* strain 536ΔPAI I or *E. coli* strain 536ΔPAI II) (**Figures 7**, **8**). Furthermore, infection with the *E. coli* strain 536ΔPAI II exhibited a more pronounced virulence-attenuated phenotype (**Figure 2**) and a greater increase in H3K9 acetylation (**Figure 8**) compared to the *E. coli* strain 536ΔPAI I, suggesting a distinct role of PAI I_536_ and PAI II_536_ in modulating host epigenetic responses and pathogenesis. This contrasting effect of selected PAIs like PAI I_536_, PAI II_536_, and PAI V_536,_ especially on H4K5 acetylation (**Figure 8**), warrants further investigation into the individual bacterial factors encoded within these islands.

The investigation of PAI I_536_ and PAI II_536_ could provide information about the role of the toxin α-hemolysin in this context. PAI I_536_ and PAI II_536_ each carry an α-hemolysin determinant. This pore-forming RTX toxin contributes to cellular toxicity by preventing acidification of LAMP1^+^ lysosomal compartments and modulating immunity through the induction of IL-1β and LDH (44). α-hemolysin can subvert the innate immune response by suppressing proinflammatory cytokine secretion and manipulating host signaling pathways, including NF-kB, MAPK, caspases and AKT (45, 46). To determine whether α-hemolysin also affects histone modifications, such as the loss of H4K5 acetylation seen in larvae infected with in *E. coli* strain 536ΔPAI I or *E. coli* strain 536ΔPAI II (**Figure 7**), we used a non-hemolytic *E. coli* 536 mutant (*E. coli* strain 536 HDM) lacking the *hly* operons located on PAI I_536_ and PAI II_536_, but in which these PAIs have otherwise been preserved. Reduced H4K5 acetylation due to the loss of PAI I_536_ or PAI II_536_ positively correlated with the loss of the two α-hemolysin operons (**Figures 8C, D**, **9A, B**). Moreover, the role of α-hemolysin in modulating the host response to UPEC infection, initially observed in larvae, was further validated in human BECs, supporting the relevance and translational value of the *G. mellonella* model (**Figure 9**). These findings demonstrate that α-hemolysin significantly influences H4K5 acetylation levels and contributes to UPEC virulence. The observed virulence attenuation in the non-hemolytic mutant 536 HDM was comparable to that seen in strains lacking either PAI I_536_ or PAI II_536_ individually, though it was less pronounced than the effect observed when both PAIs are simultaneously deleted. This suggests that additional virulence factors encoded within PAI I_536_ and PAI II_536_ also play a critical role in supporting full UPEC virulence.

Furthermore, we wanted to extend our investigations into the influence of individual virulence factors on chromatin dynamics. As it has been shown before that the K5 capsule type contributes significantly to protection of UPEC against phagocytosis (47), we analyzed how far the K15 capsule is responsible for the PAI V_536_-associated effects that we observed. H4K5 acetylation levels in *E. coli* 536ΔPAI V-infected larvae were similar to those caused by the UPEC strain 536 strain 24 h post-infection despite significantly attenuated virulence and could not be linked to expression of the K15 capsule gene cluster (**Supplementary Figures 5A, B**), because deletion of the K15 capsule operon resulted in similar virulence attenuation and H4K5 acetylation in infected larvae as deletion of the entire PAI V_536_ (compare **Figure 2B**). Furthermore, deletion of the *clbA* gene, encoding a phosphopantetheinyl transferase involved in colibactin synthesis and located within the polyketide synthase (*pks*) genomic island (PAI VI_536_), also attenuated UPEC virulence in larvae (**Supplementary Figure 6**). Our data suggest a previously unexplored contribution of individual virulence genes in shaping differential host responses and histone acetylation during infection with *E. coli* strain 536.

Histone acetylation promotes the expression of innate immunity genes, leading to the production of AMPs in cells challenged with *E. coli* (48). We found that *G. mellonella* larvae selectively express antimicrobial factors when challenged with the different PAI mutants of *E. coli* strain 536. For instance, infection with the double mutant *E. coli* 536ΔPAI I ΔPAI II mutant led to a reduced expression of proteins and peptides with strong antibacterial activity along with low level of H4K9 acetylation. This suggests a strong association between histone hypoacetylation and immune suppression (**Figures 5**, **8**). In contrast, infection with *E. coli* 536ΔPAI III or *E. coli* 536ΔPAI V mutant resulted in increased expression of antimicrobial genes (lysozyme and galiomicin) accompanied by elevated H4K5 acetylation. These findings support the role of histone acetylation in enhancing immune responses. The expression of these selected antimicrobial factors was notably upregulated in *E. coli* strain 536ΔPAI II-infected larvae in comparison to infection with the UPEC strain 536, suggesting increased host immunogenicity of bacterial factors expressed or presented upon deletion of PAI II_536_. Together, these results demonstrate that individual PAIs differentially influence both histone lysine acetylation and the expression of host antibacterial factors, thereby contributing to the complex interplay between UPEC virulence and host immune modulation.

We conclude that in the *G. mellonella* larval infection model, individual PAI deletion mutants cause similar effects as in murine infection models (17, 38, 39), which underlines the validity of the larval infection model and confirms that PAIs and the corresponding encoded virulence factors confer a selective advantage to UPEC during infection. Using complementary experimental approaches, we demonstrate that individual PAIs can selectively modulate the expression of innate immune responses as well as epigenetic processes in the host during infection. However, it remains to be investigated whether PAI-encoded factors directly or indirectly affect epigenetic mechanisms, such as histone acetylation. Alterations in chromatin dynamics of the infected host represent another largely unknown regulatory level at which the host response can be influenced during infection. Therefore, we propose using *G. mellonella* larvae as a simple and cost-effective model to study (i) the role of individual bacterial virulence factors in inducing epigenetic changes in the host, (ii) their interaction with, and (iii) their influence on the expression of epigenetic factors during infection. Selected findings from these studies can then be validated using more complex mammalian models.

## Data Availability

The raw data supporting the conclusions of this article will be made available by the authors, without undue reservation.

## Supplementary material

Supplementary Figure 1 Impact of PAIs I_536_-VI_536_ on the transcriptional activation of antimicrobial gene expression in *E. coli* strain 536-infected *G. mellonella*. The expression of the **(A, B)** IMPI, and **(C, D)** galiomicin genes was assessed in larvae at 8 h **(A, C)** and 24 h **(B, D)** post-injection with different PAI deletion mutants (536ΔPAI I - 536ΔPAI VI and 536ΔPAI I ΔPAI II) of UPEC strain 536 by quantitative real-time RT-PCR. Basal expression in infected larvae was calculated as fold-change relative to mock-injected control larvae and normalized to the 18S rRNA housekeeping gene. Statistical differences were calculated relative to infection with the UPEC strain 536 (control). Results represent mean values of at least three independent determinations ± SE (*P < 0.05; ***P < 0.0005).

Supplementary Figure 2 Impact of PAIs I_536_-VI_536_ on the transcriptional activation of antimicrobial gene expression in *E. coli* strain 536-infected *G. mellonella*. The expression of the **(A, B)** apolipophoricin-III, and **(C, D)** prophenoloxidase genes was assessed in larvae at 8 h (A, C) and 24 h **(B, D)** post-injection with different PAI deletion mutants (536ΔPAI I - 536ΔPAI VI and 536ΔPAI I ΔPAI II) of UPEC strain 536 by quantitative real-time RT-PCR. Basal expression in infected larvae was calculated as fold-change relative to mock-injected control larvae and normalized to the 18S rRNA housekeeping gene. Statistical differences were calculated relative to infection with the UPEC strain 536 (control). Results represent mean values of at least three independent determinations ± SE (*P < 0.05; **P < 0.005).

Supplementary Figure 3 Transcriptional activation of histone deacetylases (HDAC) and histone acetyltransferase (HAT) gene expression in *E. coli* strain 536-infected RT-112 cells. The expression of **(A)** HDAC2, HDAC4, HDAC9, and **(B)** KAT6A, KAT6B and KAT7 genes was assessed in RT-112 cells at 2.5 h post-injection with UPEC strain 536 by quantitative real-time RT-PCR. Basal expression in infected RT-112 cells was calculated as fold-change relative to uninfected control cells and normalized to the GAPDH rRNA housekeeping gene. Differences in gene expression were calculated relative to RT-112 cells infected with the wild-type UPEC strain 536 (control). Results represent mean values of at least three independent determinations ± SE (*P < 0.05; **P < 0.005).

Supplementary Figure 4 Impact of *E. coli* strain 536 infection on the transcriptional activation of selected HDAC and HAT genes in urinary bladder and kidney tissue of C57BL/6 mice. The expression levels of SIRT3 (HDAC) and KAT2A (HAT) were evaluated in the urinary bladder and kidney tissue 24 h post-infection by RT-PCR. Relative gene expression was calculated as fold-change relative to respective uninfected control tissues and normalized to the housekeeping gene GAPDH. Results represent mean values ± SE from two independent biological replicates for bladder samples and three for kidney samples.

Supplementary Figure 5 Contributions of the K15 capsule to virulence of the *E. coli* strain 536 in *G. mellonella*. Survival and H4K5 acetylation of larvae were assessed post-injection with UPEC strain 536 and *E. coli* strain 536ΔK15 (536Δ*kps*_K15_) **(A, B)**. Kaplan-Meier survival plots indicated reduced mortality compared to the wild-type strain. Changes in H4K5 acetylation levels was assayed 24 h post-injection using ELISA. Statistical differences were calculated relative to infection with the UPEC strain 536 (control). Results represent mean values of at least three independent determinations ± SE (*P < 0.05; **P < 0.005; ***P < 0.0005).

Supplementary Figure 6 Contributions of *clbA* to virulence of the *E. coli* strain 536 in *G. mellonella*. Kaplan-Meier survival plots of larvae after injection with UPEC strain 536Δ*clbA* showed reduced mortality compared to the wild-type strain. Larvae injected with an empty needle served as uninfected controls. Statistical differences were calculated relative to infection with the UPEC strain 536 (control). Results represent means of at least three independent determinations for 10 animals per treatment (*P < 0.05; ***P < 0.0005).

SUPPLEMENTARY TABLE 1 List of bacterial strains and plasmids used in this study.

SUPPLEMENTARY TABLE 2 List of primers used in this study.

SUPPLEMENTARY TABLE 3 Percentage growth inhibition of PAI I_536_ - VI_536_ mutants in *G. mellonella* hemolymph.
