# Supplementary figures and images for: Differential perception of virulence factors of uropathogenic Escherichia coli at the level of chromatin dynamics of infected host cells
Source: Front Immunol. 2025 Oct 6;16:1642683. doi: 10.3389/fimmu.2025.1642683 (PMC12535880; doi:10.3389/fimmu.2025.1642683)

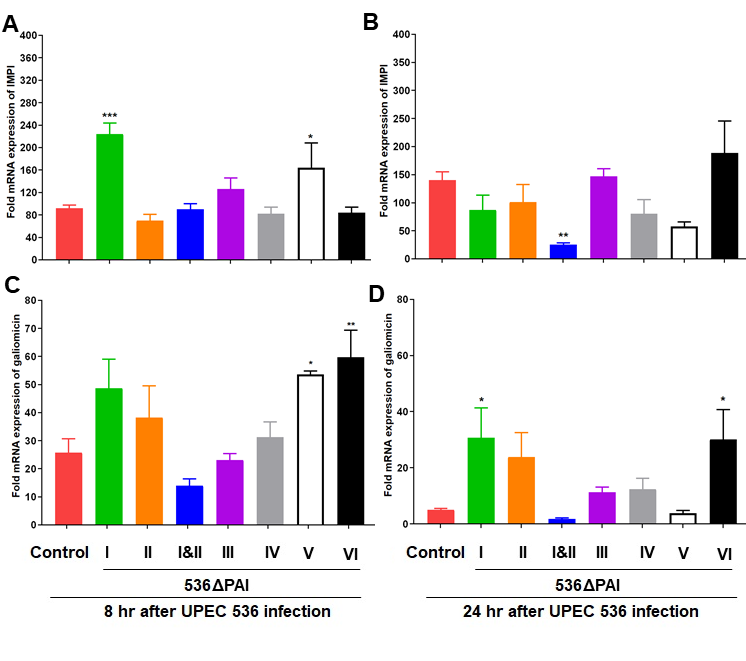

Supplement: Supplementary Figure 1 — Impact of PAIs I536-VI536 on the transcriptional activation of antimicrobial gene expression in E. coli strain 536-infected G. mellonella. The expression of the (A, B) IMPI, and (C, D) galiomicin genes was assessed in larvae at 8 h (A, C) and 24 h (B, D) post-injection with different PAI deletion mutants (536ΔPAI I - 536ΔPAI VI and 536ΔPAI I ΔPAI II) of UPEC strain 536 by quantitative real-time RT-PCR. Basal expression in infected larvae was calculated as fold-change relative to mock-injected control larvae and normalized to the 18S rRNA housekeeping gene. Statistical differences were calculated relative to infection with the UPEC strain 536 (control). Results represent mean values of at least three independent determinations ± SE (*P < 0.05; ***P < 0.0005). [file Image1.tif]

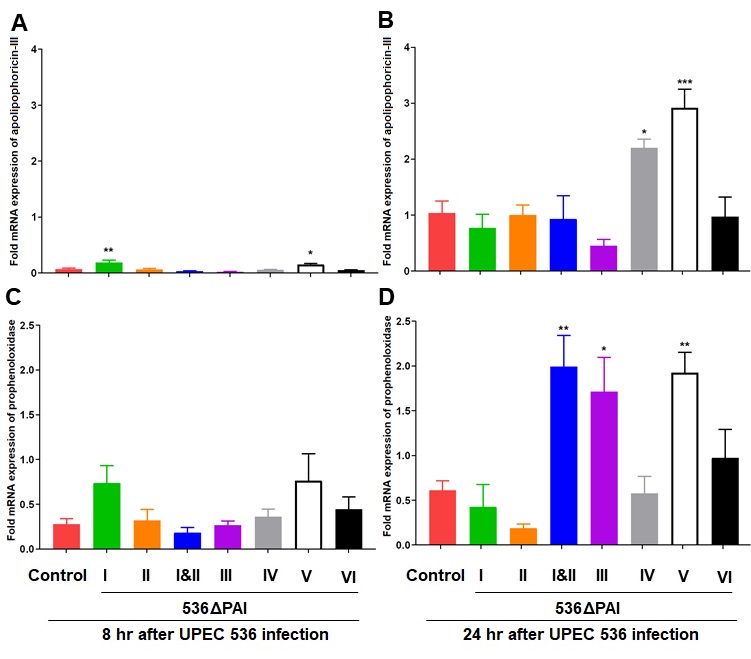

Supplement: Supplementary Figure 2 — Impact of PAIs I536-VI536 on the transcriptional activation of antimicrobial gene expression in E. coli strain 536-infected G. mellonella. The expression of the (A, B) apolipophoricin-III, and (C, D) prophenoloxidase genes was assessed in larvae at 8 h (A, C) and 24 h (B, D) post-injection with different PAI deletion mutants (536ΔPAI I - 536ΔPAI VI and 536ΔPAI I ΔPAI II) of UPEC strain 536 by quantitative real-time RT-PCR. Basal expression in infected larvae was calculated as fold-change relative to mock-injected control larvae and normalized to the 18S rRNA housekeeping gene. Statistical differences were calculated relative to infection with the UPEC strain 536 (control). Results represent mean values of at least three independent determinations ± SE (*P < 0.05; **P < 0.005). [file Image2.jpeg]

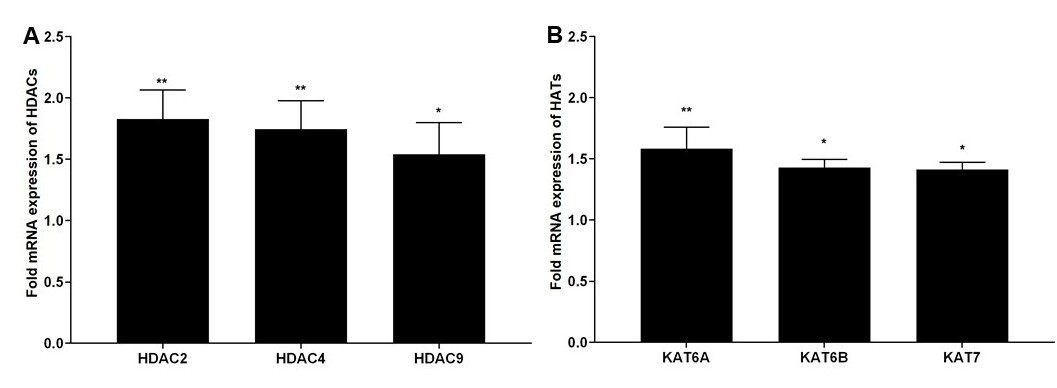

Supplement: Supplementary Figure 3 — Transcriptional activation of histone deacetylases (HDAC) and histone acetyltransferase (HAT) gene expression in E. coli strain 536-infected RT-112 cells. The expression of (A) HDAC2, HDAC4, HDAC9, and (B) KAT6A, KAT6B and KAT7 genes was assessed in RT-112 cells at 2.5 h post-injection with UPEC strain 536 by quantitative real-time RT-PCR. Basal expression in infected RT-112 cells was calculated as fold-change relative to uninfected control cells and normalized to the GAPDH rRNA housekeeping gene. Differences in gene expression were calculated relative to RT-112 cells infected with the wild-type UPEC strain 536 (control). Results represent mean values of at least three independent determinations ± SE (*P < 0.05; **P < 0.005). [file Image3.jpg]

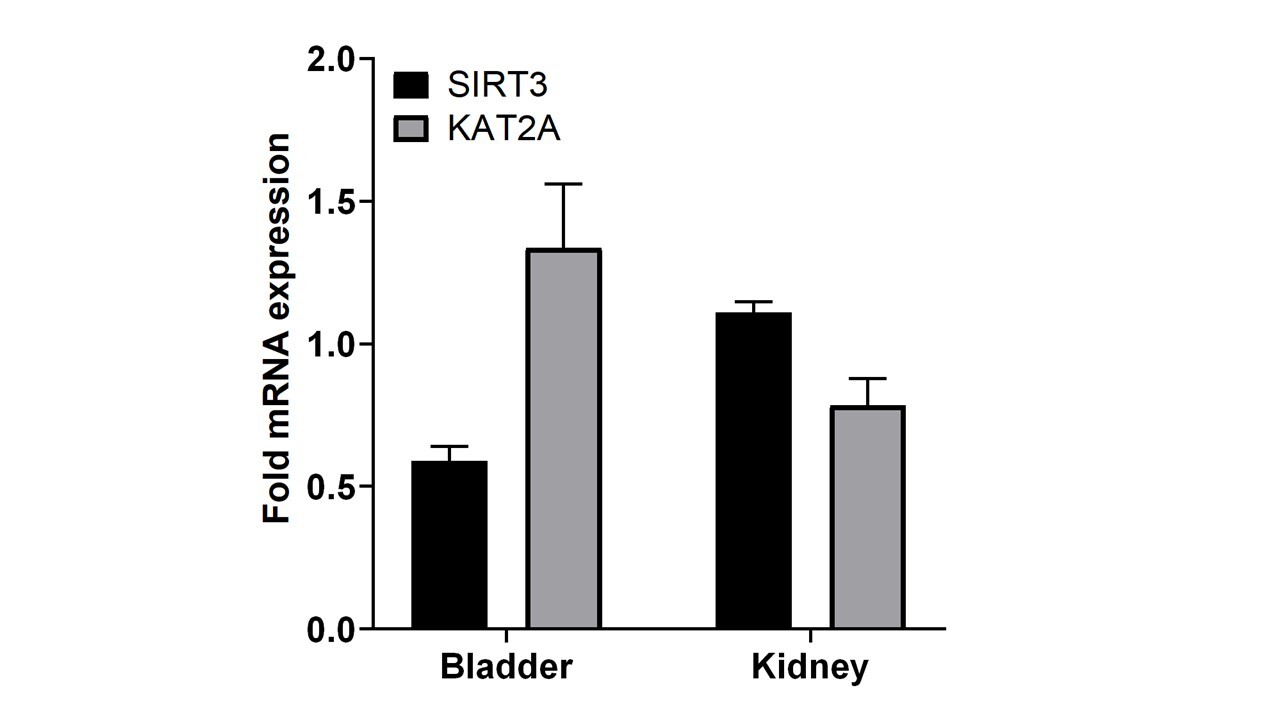

Supplement: Supplementary Figure 4 — Impact of E. coli strain 536 infection on the transcriptional activation of selected HDAC and HAT genes in urinary bladder and kidney tissue of C57BL/6 mice. The expression levels of SIRT3 (HDAC) and KAT2A (HAT) were evaluated in the urinary bladder and kidney tissue 24 h post-infection by RT-PCR. Relative gene expression was calculated as fold-change relative to respective uninfected control tissues and normalized to the housekeeping gene GAPDH. Results represent mean values ± SE from two independent biological replicates for bladder samples and three for kidney samples. [file Image4.jpg]

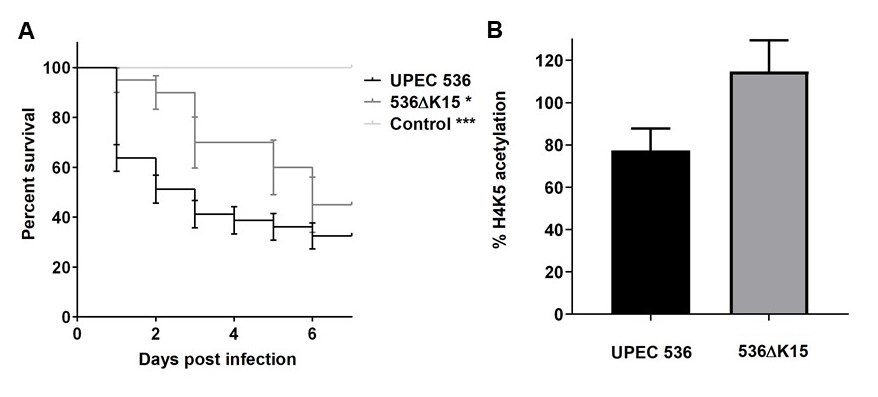

Supplement: Supplementary Figure 5 — Contributions of the K15 capsule to virulence of the E. coli strain 536 in G. mellonella. Survival and H4K5 acetylation of larvae were assessed post-injection with UPEC strain 536 and E. coli strain 536ΔK15 (536Δkps K15) (A, B). Kaplan-Meier survival plots indicated reduced mortality compared to the wild-type strain. Changes in H4K5 acetylation levels was assayed 24 h post-injection using ELISA. Statistical differences were calculated relative to infection with the UPEC strain 536 (control). Results represent mean values of at least three independent determinations ± SE (*P < 0.05; **P < 0.005; ***P < 0.0005). [file Image5.jpg]

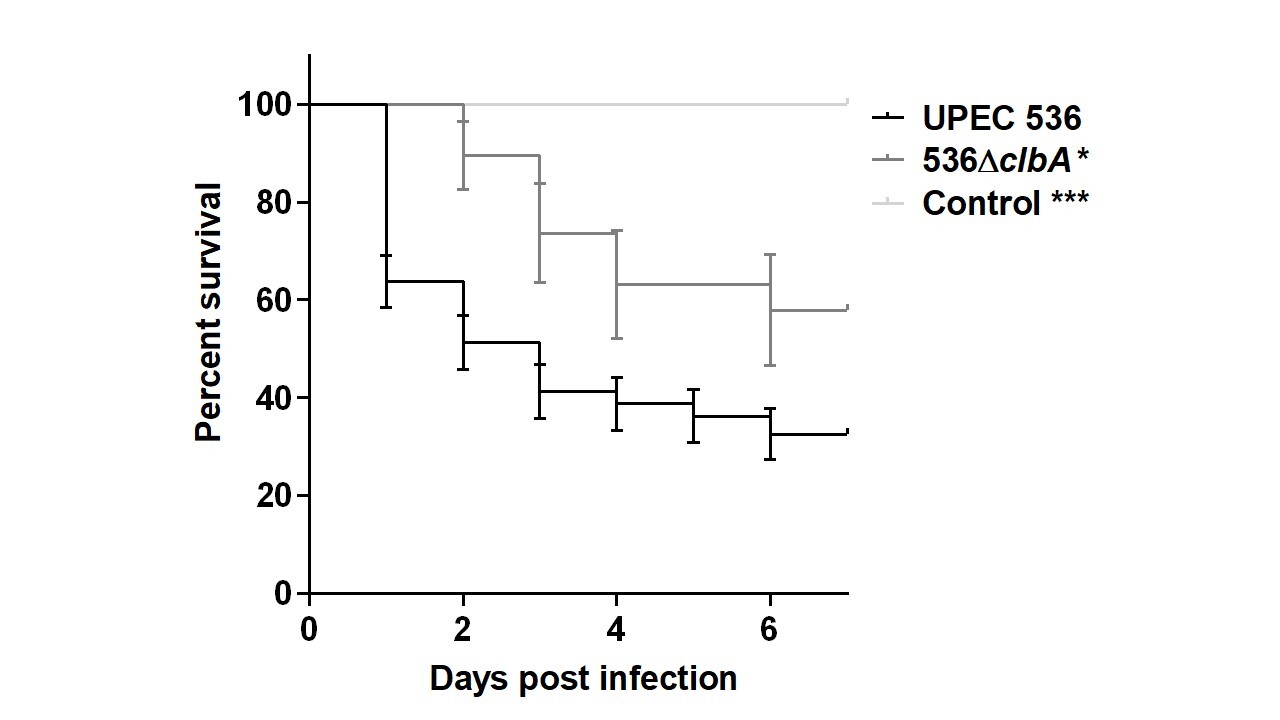

Supplement: Supplementary Figure 6 — Contributions of clbA to virulence of the E. coli strain 536 in G. mellonella. Kaplan-Meier survival plots of larvae after injection with UPEC strain 536ΔclbA showed reduced mortality compared to the wild-type strain. Larvae injected with an empty needle served as uninfected controls. Statistical differences were calculated relative to infection with the UPEC strain 536 (control). Results represent means of at least three independent determinations for 10 animals per treatment (*P < 0.05; ***P < 0.0005). [file Image6.jpg]
