# Supplementary material for: Differential perception of virulence factors of uropathogenic Escherichia coli at the level of chromatin dynamics of infected host cells
Source: Front Immunol. 2025 Oct 6;16:1642683. doi: 10.3389/fimmu.2025.1642683 (PMC12535880; doi:10.3389/fimmu.2025.1642683)
Supplement: SUPPLEMENTARY TABLE 1 — List of bacterial strains and plasmids used in this study. [file DataSheet1.pdf]

Supplementary material

Supplementary Table 1. Bacterial strains and plasmids used in this study

Strain	Genotype/Characteristics	Reference
536	pyelonephritis isolate 536, Sm^R	1
536ΔPAI I (536-114)	536, deletion of complete PAI I_{536}, Sm^R	2
536ΔPAI II (536-225)	536, deletion of complete PAI II_{536}, Sm^R	2
536 ΔPAI I ΔPAI II (536-21)	536, deletion of the complete PAI I_{536} and PAI II_{536}, Sm^R	3
536ΔPAI III	536, deletion of complete PAI III_{536}, Sm^R	4
536ΔPAI IV	536, deletion of complete PAI IV_{536}, Sm^R	4
536ΔPAI V	536, deletion of complete PAI V_{536}, Sm^R	4
536ΔPAI VI	536, deletion of complete PAI VI_{536}, Sm^R	This study
536HDM	536 Δ*hly*I, Δ*hly*II::*cat*, Sm^R	5
536Δkps_{K15}	536, deletion of the K15 *kps* locus, Sm^R, Cm^R	6
536Δ*clbA*	536, Δ*clbA*, Sm^R, Cm^R	This study
**Plasmid**	**Genotype/Characteristics**	**Reference**
pKD46	Helper plasmid for l-arabinose-inducible expression of λ-Red recombinase (*araC araB*p-*γ*-*β*-*exo*); Amp^R	7
pKD3	Template plasmid for amplification of the FRT-flanked chloramphenicol resistance cassette; FRT-*cat*-FRT; Amp^R, Cm^R	7
pKD4	Template plasmid for amplification of the FRT-flanked kanamycin resistance cassette; Amp^R, Kan^R	7
pCP20	Temperature-sensitive origin of replication, encodes Flp recombinase; Amp^R, Cm^R	8

Supplementary Table 2. List of primers used in this study

**Genes**	**Nucleotide sequence (5‘-3‘)**	**Function**
Galleria mellonella		
18S rRNA-forward<br>18S rRNA-reverse	ATGGTTGCAAAGCTGAAACT<br>TCCCGTGTTGAGTCAAATTA	RT-qPCR 18S rRNA
Lysozyme-forward<br>Lysozyme-reverse	TCCCAACTCTTGACCGACGA<br>AGTGGTTGCGCCATCCATAC	RT-qPCR lysozyme
Prophenoloxidase-forward<br>Prophenoloxidase-reverse	TGTCCAATCCGCCGCAGTTTCCTA<br>CGCCAATAAGCAAGACGGTGTTCC	RT-qPCR Prophenoloxidase
Moricin-forward<br>Moricin-reverse	GCGATCATTGCCCTCTTTAT<br>AGTGCCTTCTGTTTTTAATGTGTTC	RT-qPCR Moricin
IMPI-forward<br>IMPI-reverse	AGATGGCTATGCAAGGGATG<br>AGGACCTGTGCAGCATTTCT	RT-qPCR IMPI
Histone deacetylase 8-forward<br>Histone deacetylase 8-reverse	GATACAGTGTGGTGCGGATG<br>GCAACAAGAGCAGTGATGGA	RT-qPCR Histone deacetylase 8
Histone deacetylase complex subunit-forward<br>Histone deacetylase complex subunit-reverse	ACTTCAGGCGAGTCCATCAG<br>ACAACGAACGTTGCAGACAG	RT-qPCR Histone deacetylase complex subunit
Histone deacetylase complex subunit sap18-forward<br>Histone deacetylase complex subunit sap18-reverse	GAAACTCGACGCAAAGGAAC<br>CTCATTGGTGGAGGCATTCT	RT-qPCR Histone deacetylase complex subunit sap18

Histone acetyltransferase tip60-forward<br>Histone acetyltransferase tip60-reverse	CGCGAAATGGTAACAAACAG<br>TGGAGAGCCACATAACAACTG	RT-qPCR Histone acetyltransferase tip60
Histone acetyltransferase type b catalytic-forward<br>Histone acetyltransferase type b catalytic-reverse	CCTGAACGTTGTGGACATCA<br>CGCGCCTGTTTCTTGTTTAT	RT-qPCR Histone acetyltransferase type b catalytic
Galiomicin-forward<br>Galiomicin-reverse	GTGCGACGAATTACACCTC<br>TACTCGCACCAACAATTGAC	RT-qPCR Galiomicin
Hemolin-forward<br>Hemolin-reverse	CTCCCTCACGGAGGACAAAC<br>GCCACGCACATGTATTCACC	RT-qPCR Hemolin
Apolipophoricin III-forward<br>Apolipophoricin III-reverse	AGACTTGCACGCCATCAAGA<br>TGCATGCTGTTTGTCACTGC	RT-qPCR Apolipophoricin III
Mus musculus		
Sirtuin 3-forward<br>Sirtuin 3-reverse	ATCCCGGACTTCAGATCCCC<br>CAACATGAAAAAGGGCTTGGG	RT-qPCR Sirtuin 3
KAT2A-forward<br>KAT2A-reverse	AAGGCCAATGAAACCTGCAAG<br>CTCACAGCTACGGCACAACTC	RT-qPCR KAT2A
GAPDH-forward<br>GAPDH-reverse	TGTGTCCGTCGTGGATCTGA<br>TTGCTGTTGAAGTCGCAGGAG	RT-qPCR GAPDH
Human bladder epithelial cells (RT-112)		
HDAC2-forward<br>HDAC2-reverse	TTTTGCAGCCAGTGGTTTGT<br>TGCCTGTCCGTACTTTCCTT	RT-qPCR Histone deacetylase 2
HDAC4-forward<br>HDAC4-reverse	GATGTGGTGCTGGTGTCATC<br>AGGATCAAGCTCGTTTCCCA	RT-qPCR Histone deacetylase 4

HDAC9-forward<br><br>HDAC9-reverse	TTCAAGGTCGAAAAGCCAGC<br><br>CTCGATGACACAGCCAACAG	RT-qPCR Histone deacetylase 9
KAT6A-forward<br><br>KAT6A-reverse	GCTGGGATGGCAAACAAGAA<br><br>CAATTTGGGCAGCCTTGAGT	RT-qPCR Histone acetyltransferase KAT6A
KAT6B-forward<br><br>KAT6B-reverse	GCAGGACCAAAAGAACAGCA<br><br>AGTCTCGGCACAATCCTGAA	RT-qPCR Histone acetyltransferase KAT6B
KAT7-forward<br><br>KAT7-reverse	GGGATCATGGAATCGGACCT<br><br>GTTTTGGCACCAGGGACATT	RT-qPCR Histone acetyltransferase KAT7
GAPDH-forward<br>GAPDH-reverse	CAAATTCCATGGCACCGTCA<br>ATCTCGCTCCTGGAAGATGG	RT-qPCR GAPDH
	Escherichia coli 536	
PKS1_new<br><br>PKS1.1_noFRT_pKD3	AAAATTGGTGGTCAAATCTGGGGTCAGGTTAGTTCGATAATGGAGTGACCCCCATGTGTAGGCTGGAGCTGCTT<br><br>CAGCGCGCTACACGCCATTGCCCGGAAACCATGACCGCAGATATCTTGTTTCGTGTGCAGAATAAATAAATCCTGGTGTC	Amplification of *cat* cassette from pKD3 for insertion upstream of PAI VI_{536}
PKS2_new	AGAAGCTTTCCACGCAGGCATTATCGTAGCAGCAGTGTAAATAGACCCATTTTACATATGAATATCCTCCTTAGTTCCTA	Amplification of *npt*II from pKD4 for insertion downstream of PAI VI_{536}

PKS2.1_noFRT_pKD4	TCCTATGCAGTCCCAACTTTATCACCACGACTATCAGCTAATTCATTCGTCAAATGGGCAGCTTGCAGTGGGCTTACAT	
PKS3<br><br>PKS4	TCGGTAGAACGGCGGACTGTTAAT<br><br>ATGGACACTGCTCTAAGCGAGGTT	Proof of PAI VI_{536} deletion
clbA-del_forward<br><br>clbA-del_reverse	TTTATCACTTACAGGAATACCCCCAAAAAATCCTTATATTCTGATTACATTGTGTAGGCTGGAGCTGCTT<br><br>AAAATCAATATTATCGACGGCTCAGAAGTGTCTAGATTATCCGTGGCGATCATATGAATATCCTCCTTAGTTCCTA	Deletion of *clbA*
clbA_forward<br><br>clbA_reverse	TTTATCACTTACAGGAATACCC<br><br>TTTCAATCGACAATGCCT	Proof of *clbA* deletion

Supplementary Table 3. Percentage growth inhibition of UPEC PAIs I_{536}-VI_{536} mutants in *G. mellonella* hemolymph proteins

UPEC strains	% inhibition
536 wild-type	33%
ΔPAI I	54%
ΔPAI II	83%
ΔPAI I ΔPAI II	80%
ΔPAI III	56%
ΔPAI IV	26%
ΔPAI V	50%
ΔPAI VI	36%

Supplementary Methods

Deletion of PAI VI in *E. coli* strain 536

For deletion of the PAI VI_{536}, a *cat* cassette (amplified from pKD3) and an *npt*II cassette (amplified from pKD4) were first amplified with the primer pairs PKS1_new/PKS1.1_noFRT_pKD3 and PKS2_new/PKS2.1_noFRT_pKD4 (see Supplementary Table S2). The resulting PCR products chromosomally inserted by pKD46-mediated recombineering upstream and downstream of the PAI VI_{536}, respectively (Datsenko & Wanner, 2000). Chloramphenicol- and kanamycin-resistant double mutants were selected in which the PAI VI_{536} was now flanked by the two resistance cassettes and FRT sites. PAI VI_{536} was deleted by the activity of the Flp recombinase after transformation with pCP20 (Cherepanov & Wackernagel, 1995) via the flanking FRT sequences. The loss of PAI VI_{536} was verified by PCR with the oligonucleotide pair PKS3/PKS4 (see Supplementary Table 2) and subsequent sequencing of the PCR product.

Deletion of *clbA* in *E. coli* strain 536

The *clbA* deletion mutant of *E. coli* 536 was constructed by pKD46-mediated recombineering replacing the clbA gene by a *npt*II cassette amplified with primer pair clbA-del_for/clbA-del_rev from pKD4 (Datsenko & Wanner, 2000). Afterwards, the kanamycin resistance cassette was deleted via the flanking FRT sequences by the activity of the Flp recombinase after transformation with pCP20 (Cherepanov & Wackernagel, 1995). The loss of *clbA* was verified by PCR with the oligonucleotide pair clbA_for/clbA_rev (see Supplementary Table 2) and subsequent sequencing of the PCR product.

Ascending urinary tract infection model

Female specific pathogen-free C57BL/6JOlaHsd mice (10–12 weeks old; 17–23 g) were obtained from Envigo RMS GmbH (Düsseldorf, Germany) and used for all experimental procedures. All animal experiments were conducted in accordance with the German Animal Welfare Act (Tierschutzgesetz) and the institutional guidelines for the care and use of laboratory animals. The study was approved by

the regional regulatory authority (Approval No. 81-02.04.2022.A365). Mice were housed in a specific pathogen-free facility with ad libitum access to food and water. For infection experiments, mice were transurethrally inoculated with *Escherichia coli* strain 536 (1 × 10^{10} CFU/mL) as previously described (9). Animals were euthanized 24 hours post-infection.

To assess gene expression in the urinary bladder and kidneys, these organs were aseptically harvested, gently rinsed with 0.9% NaCl solution, and mechanically homogenized in TRIreagent. Total RNA was isolated from the bladder and kidney tissues using phenol-chloroform. Purified total RNA was quantified using a Nandrop spectrophotometer (Thermo Fisher Scientific, Dreieich, Germany) and a 2100 Bioanalyzer (Agilent Technologies, Waldbronn, Germany). The 260/280 and 260/230 absorbance ratios for RNA (> 2.0) were determined for samples used for RT-PCR analysis. Complementary DNA (cDNA) was generated from total RNA of UPEC strain *E. coli* 536-infected and uninfected murine urinary bladders and kidneys using the First Stand cDNA synthesis kit (Thermo Fisher Scientific, Darmstadt, Germany). Spectrophotometry was used to estimate the cDNA concentration. Using the SsoAdvanced™ Universal Inhibitor-Tolerant SYBR® Green supermix, quantitative real-time RT-PCR was carried out using the CFX 96 real-time PCR system (Bio-Rad Laboratories, Inc., Feldkirchen, Germany). The primers listed in Supplementary Table S2 were used to determine the expression levels of sirtuins and KAT2A using 50 ng (for kidneys) and 200 ng (bladders) of cDNA per reaction. An initial stage of denaturation at 95 °C for 10 min was followed by 39 cycles consisting of a denaturation step at 95 °C for 15 s, primer annealing at 56 °C for 15 s, and primer extension at 72 °C for 15 s.

References

1. Berger H, Hacker J, Juarez A, Hughes C, Goebel W. Cloning of the chromosomal determinants encoding hemolysin production and mannose-resistant hemagglutination in *Escherichia coli*. *J Bacteriol*. 1982 152:1241–1247. doi: 10.1128/jb.152.3.1241-1247.1982.

2. Knapp S, Hacker J, Then I, Müller D, Goebel W. Multiple copies of hemolysin genes and associated sequences in the chromosome of uropathogenic *Escherichia coli* strains. *J Bacteriol*. 1984 159: 1027–1033. doi: 10.1128/jb.159.3.1027-1033.1984.

3. Hacker J, Knapp S, Goebel W. Spontaneous deletions and flanking regions of the chromosomally inherited hemolysin determinant of an *Escherichia coli* O6 strain. *J Bacteriol*. 1983 154:1145-1152. doi: 10.1128/jb.154.3.1145-1152.1983.

4. Brzuszkiewicz E, Brüggemann H, Liesegang H, Emmerth M, Olschläger T, Nagy G, et al. How to become a uropathogen: comparative genomic analysis of extraintestinal pathogenic *Escherichia coli* strains. *Proc Natl Acad Sci U S A*. (2006) 103:12879-12884. doi: 10.1073/pnas.0603038103.

5. Nagy G, Altenhoefer A, Knapp O, Maier E, Dobrindt U, Blum-Oehler G, et al. Both alpha-haemolysin determinants contribute to full virulence of uropathogenic *Escherichia coli* strain 536. *Microbes Infect*. 2006 8:2006-2012. doi: 10.1016/j.micinf.2006.02.029.

6. Schneider G, Dobrindt U, Brüggemann H, Nagy G, Janke B, Blum-Oehler G, et al. The pathogenicity island-associated K15 capsule determinant exhibits a novel genetic structure and correlates with virulence in uropathogenic *Escherichia coli* strain 536. *Infect Immun*. 2004 72:5993-6001. doi: 10.1128/IAI.72.10.5993-6001.2004.

7. Datsenko KA, Wanner BL. One-step inactivation of chromosomal genes in *Escherichia coli* K-12 using PCR products. *Proc Natl Acad Sci U S A*. 2000 97:6640-6645. doi: 10.1073/pnas.120163297.

8. Cherepanov PP, Wackernagel W. Gene disruption in *Escherichia coli*: TcR and KmR cassettes with the option of Flp-catalyzed excision of the antibiotic-resistance determinant. *Gene*. 1995 158:9-14. doi: 10.1016/0378-1119(95)00193-a.

9. Bielaszewska M, Schiller R, Lammers L, Bauwens A, Fruth A, et al. Heteropathogenic virulence and phylogeny reveal phased pathogenic metamorphosis in *Escherichia coli* O2:H6. *EMBO Mol Med*. 2014 6:347-57. doi: 10.1002/emmm.201303133.
